# An Efficient Screen for Cell-Intrinsic Factors Identifies the Chaperonin CCT and Multiple Conserved Mechanisms as Mediating Dendrite Morphogenesis

**DOI:** 10.3389/fncel.2020.577315

**Published:** 2020-09-25

**Authors:** Ying-Hsuan Wang, Zhao-Ying Ding, Ying-Ju Cheng, Cheng-Ting Chien, Min-Lang Huang

**Affiliations:** ^1^Department of Biomedical Sciences, National Chung Cheng University, Chiayi, Taiwan; ^2^Institute of Molecular Biology, Academia Sinica, Taipei, Taiwan

**Keywords:** CCT chaperonin, microtubule, dendrite morphogenesis, genetic screen, *Drosophila*

## Abstract

Dendritic morphology is inextricably linked to neuronal function. Systematic large-scale screens combined with genetic mapping have uncovered several mechanisms underlying dendrite morphogenesis. However, a comprehensive overview of participating molecular mechanisms is still lacking. Here, we conducted an efficient clonal screen using a collection of mapped P-element insertions that were previously shown to cause lethality and eye defects in *Drosophila melanogaster*. Of 280 mutants, 52 exhibited dendritic defects. Further database analyses, complementation tests, and RNA interference validations verified 40 P-element insertion genes as being responsible for the dendritic defects. Twenty-eight mutants presented severe arbor reduction, and the remainder displayed other abnormalities. The intrinsic regulators encoded by the identified genes participate in multiple conserved mechanisms and pathways, including the protein folding machinery and the chaperonin-containing TCP-1 (CCT) complex that facilitates tubulin folding. Mutant neurons in which expression of CCT4 or CCT5 was depleted exhibited severely retarded dendrite growth. We show that CCT localizes in dendrites and is required for dendritic microtubule organization and tubulin stability, suggesting that CCT-mediated tubulin folding occurs locally within dendrites. Our study also reveals novel mechanisms underlying dendrite morphogenesis. For example, we show that *Drosophila* Nogo signaling is required for dendrite development and that Mummy and Wech also regulate dendrite morphogenesis, potentially via Dpp- and integrin-independent pathways. Our methodology represents an efficient strategy for identifying intrinsic dendrite regulators, and provides insights into the plethora of molecular mechanisms underlying dendrite morphogenesis.

## Introduction

Appropriate dendritic morphology is critical for neurons to build circuits and to receive and integrate stimulations. Aberrant dendritic arborization impairs circuit function and is correlated with neurological and neurodevelopmental disorders, such as schizophrenia, Down’s syndrome, fragile X syndrome, and autism spectrum disorders (Jan and Jan, 2010). Various intrinsic factors are required for dendrite morphogenesis (Puram and Bonni, 2013; Dong et al., 2015). For instance, transcription factors can specify neuronal types and direct dendritic morphology, cytoskeletal and motor proteins provide structural support and are the basis for intracellular transport of cargos that control dendrite growth, and secretory and endocytic pathways can shape dendritic arborization. However, the overall program that establishes dendritic arbors remains incompletely understood. Thus, identifying the contributory factors and their associated pathways is paramount to fully resolving how neurons develop and the pathogenesis of neurological disorders.

We selected *Drosophila* peripheral dendritic arborization (da) neurons as an elegant model system as the four different classes (I–IV) of those neurons display distinct and characteristic dendritic morphologies (Grueber et al., 2002). Given evolutionary conservation of the pathways regulating dendritic morphology, this model system has been widely used as a platform for large-scale screens to identify factors involved in dendrite morphogenesis (Puram and Bonni, 2013; Valnegri et al., 2015). For example, RNA interference (RNAi) screens have identified dendrite regulators for class I da (c1da) and class IV da (c4da) neurons (Parrish et al., 2006; Olesnicky et al., 2014). However, due to the propensity of off-target effects from RNAi screening, additional validation is needed to confirm the specific genes involved (Kulkarni et al., 2006; Ma et al., 2006). Unbiased ethyl methanesulfonate (EMS)-based forward genetic screens are an alternative approach for identifying factors that regulate the dendritic morphology of da neurons (Gao et al., 1999; Ye et al., 2007; Zheng et al., 2008). Mapping mutations identified from EMS-based screens is tedious, and early lethality of homozygous mutants prevent analyses of defective dendrites. To overcome this latter problem, mosaic clones for specific mutations have to be generated to observe morphological dendritic defects. However, the conventional heat-shock flippase (FLP) approach to generate clones is labor-intensive and the clone recovery rate is low (Grueber et al., 2002). Hence, novel genetic screens must be devised to efficiently generate neuronal clones, enabling identification of the genes involved in dendrite morphogenesis.

Here, we integrated a variety of screening systems to devise an effective protocol for screening dendrite regulators. Our genetic screen is based on the mosaic analysis with a repressible cell marker (MARCM) system (Lee and Luo, 1999), induced by sensory neuron-specific FLP combined with a c4da neuronal marker (Shimono et al., 2014). We used this system to screen a collection of mapped P-element insertions that are known to cause lethality and morphological defects of the eye (Chen et al., 2005; Call et al., 2007). Our approach enabled efficient identification of genes involved in dendrite morphogenesis, which were further validated by RNAi-based analyses. We screened more than 200 P-element mutants, and identified 40 genes that encode significant components of various protein complexes or that are involved in distinct pathways.

We further focused our study on chaperonin-based regulation of dendrite morphogenesis. We found that mutations of subunits of chaperonin-containing TCP-1 (CCT, also named TCP1-ring complex, TRiC) severely retarded growth of dendritic arbors. Eukaryotic CCT is a hetero-oligomeric complex of two stacked rings, each consisting of eight distinct subunits (CCT1–8) (Yaffe et al., 1992; Vinh and Drubin, 1994; Yam et al., 2008). The CCT chaperonin facilitates protein folding, including the cytoskeletal components tubulin and actin. Our data suggest that CCT localizes in dendrites and participates in dendrite morphogenesis most likely by regulating local microtubule biogenesis. Moreover, further validation experiments demonstrate the essentiality of the mechanisms or pathways we identified for correct dendritic development. Our study portrays an appropriate strategy for identifying dendrite regulators, and provides insights into how dendrites develop.

## Results and Discussion

### An Efficient Screen Identifies Cell-Intrinsic Factors Required for Dendritic Arborization

#### SOP-FLP-Based MARCM Screen and Validation of P-Insertion Genes

**FIGURE 1:**
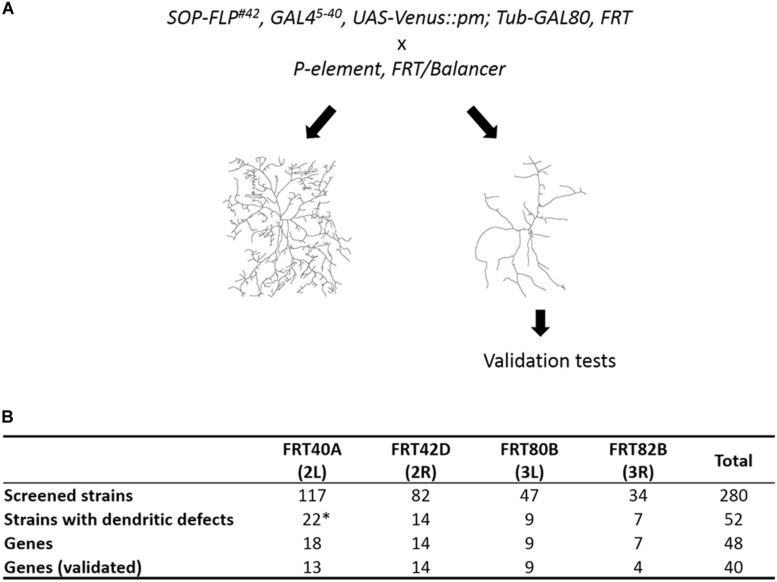
Summary of our SOP-FLP-based MARCM screen. **(A)** Scheme for generating MARCM clones. P-insertion mutants were crossed individually to SOP-FLP-based MARCM-ready fly stocks. The c4da neurons were imaged and those P-insertion mutants exhibiting dendritic defects were further validated. **(B)** Table showing the numbers of P-insertion mutants and genes identified for each chromosome arm. *Two genes have two P-element alleles, and two P-element insertion sites are unknown (see Supplementary Table S1).

To identify cell-intrinsic factors required for dendrite morphogenesis, we conducted a MARCM genetic screen employing the *SOP-FLP* transgene that drives FLP expression in sensory organ precursors (Shimono et al., 2014). The screen was conducted on the BruinFly collection of lethal P-element insertions^1^, whose clonal *Drosophila* mutants display severely defective eye morphologies (Chen et al., 2005; Call et al., 2007). The SOP-FLP-based MARCM-ready *Drosophila* also bear the *UAS-Venus::pm* transgene for labeling dendritic membranes, enabling efficient F1 screening of these P-insertion strains (Figure 1A). As the c4da neuron displays the most complex dendritic pattern among the four types of da neurons, we chose the ddaC neuron of the c4da type for phenotypic assessment (Grueber et al., 2002). The ddaC neuron exhibits complex dendritic arbors covering the dorso-abdominal segment of third instar larva. As anticipated, a high frequency of P-insertion mutants (52 of 280) presented detectable dendritic defects, including significant reduction in dendritic branches or fields, or alteration in dendritic patterns (Figure 1B and Supplementary Table S1). By subtracting allelic and unmapped P-insertions, we conclude that 48 different genes were disrupted by these P-insertions (Figure 1B).

We employed four strategies to confirm that disruption of these P-insertion genes indeed caused the observed phenotypes (Supplementary Table S2). Firstly, we searched the FlyBase database and found that 24 of our 48 P-insertion mutants had been studied previously, and the respective P-insertions had been mapped to corresponding genes and were shown to cause the mutant phenotypes. Secondly, we performed complementation tests using different lethal alleles or chromosomal deficiency alleles and confirmed that 28 of our 48 P-element lines harbor lethal insertions. Thirdly, we endeavored to individually knockdown the 48 P-insertion genes in c4da neurons by means of RNAi to recapitulate dendritic phenotypes and confirmed that 21 P-insertion genes yielded dendritic defects. Fourthly, we used MARCM clones generated for secondary lethal alleles to confirm that 5 P-insertion genes are involved in dendrite morphogenesis. Together, our RNAi- and MARCM-based experiments verified that 24 P-insertion genes are responsible for the dendritic defects we observed.

Eight P-insertion strains failed to pass the validation test for both lethality and dendritic defects (Supplementary Table S2). The loss-of-function allele *ab*^*k02807*^ exhibited retarded dendrite growth, inconsistent with a previous finding that *abrupt* (*ab*) is not required for c4da dendrite development (Li et al., 2004; Sugimura et al., 2004). Thus, in this case, the dendritic defect could be caused by a second-site mutation. The remaining seven P-insertion strains—*rgr*^*k02605*^, *SCAR*^*k03107*^, *trx*^*j14A6*^, *CG5446*^*KG06435*^, *CG15141*^*KG06005*^, *CG42327*^*KG05924*^, and *TkR99D*^*s2222*^–were complemented by other lethal alleles or deficiencies. RNAi knockdown of these genes also failed to recapitulate dendritic phenotypes. Accordingly, we concluded that mutations in these eight genes are not responsible for the observed dendritic defects from our MARCM clonal analysis (Figure 1B). Below, we report on the remaining 40 genes whose mutations caused dendritic defects.

#### Dendritic Defects Induced by P-Insertions

**FIGURE 2:**
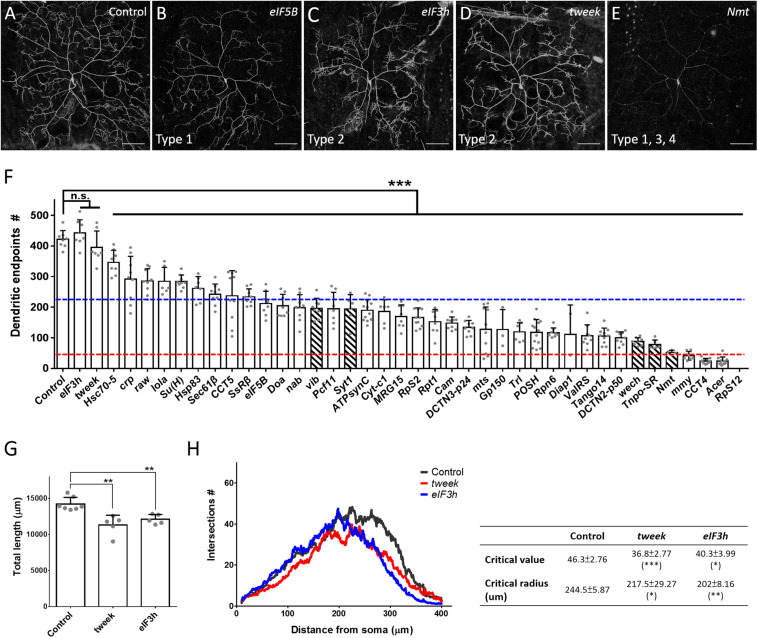
Phenotypic analyses of the P-insertion mutants. **(A–E)** Representative images for c4da MARCM clones of control **(A)**, *eIF5B*^*KG09489*^ **(B)**, *eIF3h*^*k09003*^ **(C)**, *tweek*^*EY02585*^ **(D)**, and *Nmt*^*j1C7*^ **(E)** neurons. The categories of dendritic defects for each mutant neuron were indicated. Scale bars = 100 μm. **(F)** Numbers of dendritic endpoints of c4da MARCM mutant neurons, with blue and red lines marking 50% and 10% of branch numbers relative to control, respectively. Hatched bars indicate mutants with faint *Venus::pm* signal intensities. The P-insertion mutant alleles used for quantification are listed in Table 1. **(G,H)** Total dendritic length **(G)** and Sholl analysis **(H)** for c4da MARCM clones of control, *tweek* or *eIF3h* neurons. The table (right panel of **H**) shows values for maximal dendritic branch intersections (critical value) and corresponding radii (critical radius). *n* = 5 neurons per genotype. Student’s *t*-test *P* values (*P < 0.05; **P < 0.01; ***P < 0.001).

**FIGURE 3:**
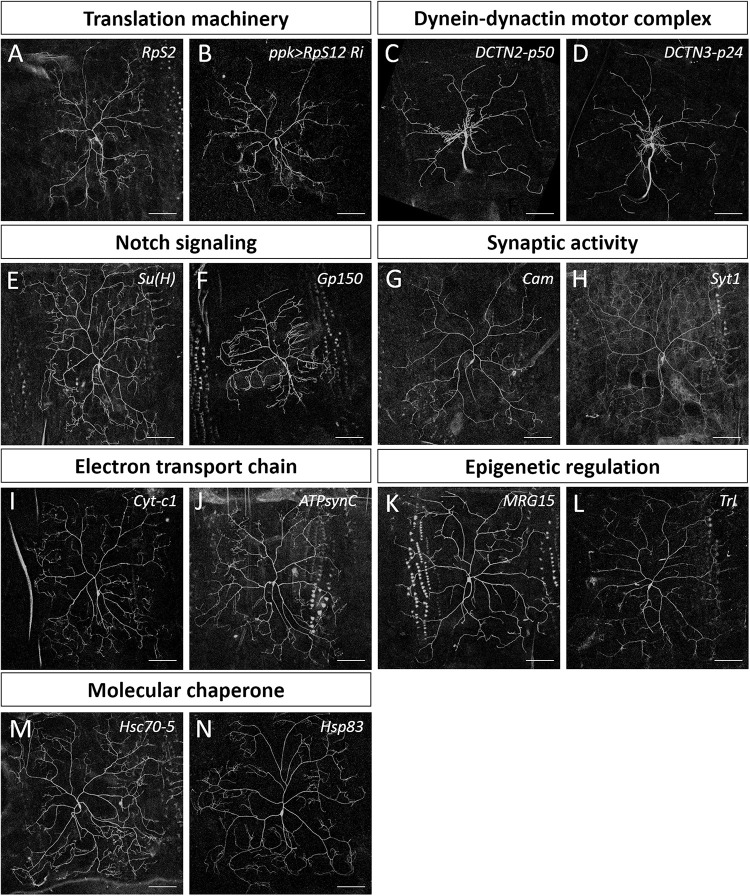
Dendrite regulators in conserved complexes or pathways. **(A,C–N)** Representative c4da MARCM clones for indicated mutants with alleles *RpS2*^*k01215*^ **(A)**, *DCTN2-p50^*k16109*^* **(C)**, *DCTN3-p24^*k14618*^* **(D)**, *Su(H)^*k07904*^* **(E)**, *Gp150*^*k11120*^ **(F)**, *Cam*^*k04213*^ **(G)**, *Syt1*^*k05909*^ **(H)**, *Cyt-c1^*KG05986*^* **(I)**, *ATPsynC*^*KG01914*^ **(J)**, MRG15*^*j6A3*^* **(K)**, *Trl*^*s2325*^ **(L)**, *Hsc70-5^*k04907*^* **(M)**, and *Hsp83*^*j5C2*^ **(N)**. **(B)** The mCD8::GFP-labeled dendrite of a c4da neuron with *RpS12-RNAi* knockdown by *ppk-GAL4*. Scale bars = 100 μm.

The dendritic phenotypes of the remaining 40 P-insertion mutants were diverse, but could be categorized into four classes (Table 1). The first of these classes, representing a reduction in dendritic branches (as assessed by counting dendritic endpoints), was the most frequently observed phenotype, which was detected in 38 P-insertion lines, as shown by the representative *eIF5B* mutant clones (Figures 2A,B). Of these 38 lines, 28 (74%) exhibited a severe reduction of more than 50% dendritic branches (Figure 2F, dashed blue line represents the 50% threshold). In the particularly extreme cases of *mmy*, *CCT4*, and *Acer* mutant neurons, dendritic branch numbers were reduced to less than one-tenth of the control (Figure 2F, dashed red line represents the 10% threshold). None of the mutant neurons we investigated exhibited increased dendritic branching. The second class, abnormal patterns of dendritic arborization despite normal branch numbers, was manifested in *tweek* and *eIF3h* mutant neurons (Figures 2C,D,F). Numbers of dendritic endpoints were unaffected in both of these mutants, but total dendritic length was reduced (Figure 2G), and Sholl analyses revealed reduced dendritic complexity in distal regions (Figure 2H). The third class of phenotypes, faint *Venus::pm* signal intensities in dendrites, was observed in five of the branch-reduction mutant lines (shown as hatched bars in Figure 2F). For this class, the reduced numbers of dendritic branches was not due to diminished *Venus::pm* signal intensities because branch numbers were scored under enhanced intensities. For example, *vib* and *Syt1* mutant neurons still retained close to half the number of dendritic branches as control neurons, despite their reduced *Venus::pm* signal intensities. Among the third class of mutants, *Nmt* mutant neurons exhibited an extreme reduction of both *Venus::pm* signal intensity and dendritic branching (Figures 2E,F). The reduction of signal could be caused by weaker expression from the GAL4 driver or posttranscriptional mechanisms regulating the Venus::pm protein synthesis and transport, which were not further studied here. Finally, the fourth class of mutant neurons (including *RpS12* and another three mutants) exhibited a low frequency of MARCM-based recovery neurons (fewer than 5 neurons from more than 150 screened larvae). Although *RpS12* mutant neurons were not recovered from the third instar larval stage, their dendritic defect was validated by respective RNAi knockdown (Figures 2F, 3B).

**TABLE 1:** List of genes identified in the MARCM screen of P-insertion lines.

Symbol	Name	Human ortholog^a^	Phenotype^b^	Allele
*Acer*	*Angiotensin-converting enzyme-related*	*ACE*	1	*Acer*^*k07704*^
*ATPsynC*	*ATP synthase, subunit C*	*ATP5G2*	1	*ATPsynC*^*KG01914*^
*Cam*	*Calmodulin*	*CALM1*	1	*Cam*^*k04213*^
*CCT4*	*Chaperonin containing TCP1 subunit 4*	*CCT4*	1	*CCT4*^*KG09280*^
*CCT5*	*Chaperonin containing TCP1 subunit 5*	*CCT5*	1	*CCT5*^*k06005*^
*crp*	*Cropped*	*TFAP4*	1	*crp*^*k00809*^
*Cyt-c1*	*Cytochrome c1*	*CYC1*	1	*Cyt-c1^*KG05986*^*
*DCTN2-p50*	*Dynactin 2, p50 subunit*	*DCTN2*	1	*DCTN2-p50^*k16109*^*
*DCTN3-p24*	*Dynactin 3, p24 subunit*	*DCTN3*	1	*DCTN3-p24^*k14618*^*
*Diap1*	*Death-associated inhibitor of apoptosis 1*	*XIAP*	1,4	*Diap1*^*j5C8*^
*Doa*	*Darkener of apricot*	*CLK2*	1	*Doa*^*s2784*^
*eIF3h*	*Eukaryotic translation initiation factor 3 subunit h*	*EIF3H*	2	*eIF3h*^*k09003*^
*eIF5B*	*Eukaryotic translation initiation factor 5B*	*EIF5B*	1	*eIF5B*^*KG09489*^
*Gp150*	*Gp150*		1,4	*Gp150*^*k11120b*^
*Hsc70-5*	*Heat shock protein cognate 5*	*HSPA9*	1	*Hsc70-5^*k04907*^*
*Hsp83*	*Heat shock protein 83*	*HSP90AB1*	1	*Hsp83*^*j5C2*^
*lola*	*Longitudinals lacking*		1	*lola*^*k09901*^
*mmy*	*mummy*	*UAP1*	1	*mmy*^*KG08617*^
*MRG15*	*MORF-related gene 15*	*MORF4L1*	1	*MRG15*^*j6A3*^
*mts*	*Microtubule star*	*PPP2CA*	1	*mts*^*s5286*^
*nab*	*Nab*	*NAB2*	1	*nab*^*KG07676*^
*Nmt*	*N-myristoyl transferase*	*NMT1*	1,3,4	*Nmt*^*j1C7*^
*Pcf11*	*Protein 1 of cleavage and polyadenylation factor 1*	*PCF11*	1	*Pcf11*^*k08015*^
*POSH*	*Plenty of SH3s*	*SH3RF3*	1	*POSH*^*k15815*^
*raw*	*Raw*		1	*raw*^*k01021*^
*Rpn6*	*Regulatory particle non-ATPase 6*	*PSMD11*	1	*Rpn6*^*k00103*^
*RpS12*	*Ribosomal protein S12*	*RPS12*	4	*RpS12*^*s2783*^
*RpS2*	*Ribosomal protein S2*	*RPS2*	1	*RpS2*^*k01215*^
*Rpt1*	*Regulatory particle triple-A ATPase 1*	*PSMC2*	1	*Rpt1*^*k11110*^
*Sec61*β	*Sec61 β subunit*	*SEC61*β	1	*Sec61β^*k03307*^*
*SsR*β	*Signal sequence receptor* β	*SSR2*	1	*SsRβ^*s1939*^*
*Su(H)*	*Suppressor of Hairless*	*RBPJ*	1	*Su(H)^*k07904*^*
*Syt1*	*Synaptotagmin 1*	*SYT1*	1,3	*Syt1*^*k05909*^
*Tango14*	*Transport and Golgi organization 14*	*NUS1*	1	*Tango14*^*k00619*^
*Tnpo-SR*	*Transportin-Serine/Arginine rich*	*TNPO3*	1,3	*Tnpo-SR^*KG04870*^*
*Trl*	*Trithorax-like*		1	*Trl*^*s2325*^
*tweek*	*Tweek*	*KIAA1109*	2	*tweek*^*EY02585*^
*ValRS*	*Valyl-tRNA synthetase*	*VARS*	1	*ValRS*^*k14804*^
*vib*	*Vibrator*	*PITPNB*	1,3	*vib*^*j7A3*^
*wech*	*Wech*	*TRIM71*	1,3	*wech*^*k08815*^

*^*a*^Orthologs with DIOPT v6 score > 2 are shown. ^*b*^Dendritic defects were categorized into four types: 1. Significant reduction in branch numbers; 2. Abnormal dendritic patterns with normal branch numbers; 3. Dramatic reduction in *Venus::pm* signal; 4. Low MARCM clone frequency (<5 clones recovered from more than 150 larvae).*

### Conserved Complexes and Pathways Regulate Dendrite Development

Our screening strategy identified mutations in the genes *lola* and *raw* as being responsible for defective dendritic morphology, both of which have been studied previously in da neurons (Ferreira et al., 2014; Lee et al., 2015), evidencing the effectiveness of our approach. We found that 90% of the genes we identified from our screen have human orthologs, indicative of evolutionary conservation (Table 1). Notably, three housekeeping genes we identified from our screen—*Pcf11*, *Diap1*, and *Nmt*—are involved in cell growth and survival, individual loss of which causes growth retardation and apoptosis (Eisenberg and Levanon, 2013). The housekeeping functions of these particular genes might explain the severe dendrite reductions and low clonal recovery rates of the respective mutants (Table 1 and Supplementary Figures S1A–C). We also identified the transcription factor Crp (King-Jones et al., 1999), the transcription co-factor Nab (Terriente Felix et al., 2007), and the peptidase Acer (Coates et al., 2000) as being dendrite regulators (Supplementary Figures S1D–F). Further investigation of these proteins is required to reveal the pathways by which they regulate dendritic arborization.

We categorized the remaining 32 dendrite regulators according to known molecular mechanisms and pathways (Table 2). Some of these regulators are known to be required for c4da dendrite development, including components of the translation initiation complex, eIF3h and eIF5B, as well as the 40S ribosomal subunits RpS2 and RpS12 (Figures 2B,C,F, 3A,B), which confirms involvement of the translation machinery in dendritic development (Olesnicky et al., 2014; Nanda et al., 2018). Furthermore, mutants for the dynactin cofactors DCTN2-p50 or DCTN3-p24 displayed the “proximal bushy” phenotype (Figures 2F, 3C,D), observed previously for dynein subunit mutants (Satoh et al., 2008; Zheng et al., 2008), implicating the dynein-dynactin motor complex in dendrite morphogenesis.

**TABLE 2:** Mechanisms and signaling pathways revealed in the screen.

Mechanisms/pathways	Dendrite regulators identified in this study
Translation machinery*	eIF3h, eIF5B, RpS2, RpS12
Dynein-dynactin motor complex*	DCTN2-p50, DCTN3-p24
Notch signaling*	Su(H), Gp150
Synaptic activity*	Cam, Syt1
Electron transport chain*	Cyt-c1, ATPsynC
Epigenetic regulation*	MRG15, Trl
CCT chaperonin*	CCT4, CCT5
Molecular chaperone*	Hsc70-5, Hsp83
PP2A holoenzyme	Mts
26S proteasome*	Rpt1, Rpn6
ER translocon*	Sec61β, SsRβ
Aminoacyl-tRNA synthetase	ValRS
Nogo signaling*	Tango14, POSH
Regulation of SR proteins*	Tnpo-SR, Doa
Regulation of phosphoinositides*	Vib, Tweek
Dpp-independent function of Mmy	Mmy
Integrin-independent function of Wech	Wech

*Mechanisms or signal pathways for which two or more dendrite regulators were identified from our screen.

Some of the molecular mechanisms/pathways we identified are known to be required in mammalian dendrite development. For example, the Notch signaling pathway regulates dendritic morphology in cultured rat neurons and in maturing mouse hippocampal neurons (Redmond et al., 2000; Breunig et al., 2007). We observed dendritic growth defects in mutants for the Notch pathway components Su(H) and Gp150 (Figures 2F, 3E,F). Synaptic activity participates in mammalian dendrite growth and patterning (Wong and Ghosh, 2002; Chen and Ghosh, 2005). Accordingly, we found that mutants of the calcium-binding protein Cam and the calcium-sensitive synaptic vesicle fusion protein Syt1 also displayed dendritic defects (Figures 2F, 3G,H). Similarly, the electron transport chain in mitochondria is crucial for dendrite morphogenesis of hippocampal neurons (Oruganty-Das et al., 2012), and we identified Cyt-c1 of Complex III and ATPsynC of Complex V of that electron transport chain as being regulators of dendritic arborization (Figures 2F, 3I,J). Epigenetic mechanisms are known to regulate dendritic morphology of mammalian neurons (Smrt and Zhao, 2010). Moreover, we found that the Polycomb antagonist MRG15 and Trl of the Trithorax complex are essential for normal dendritic arborization (Figures 2F, 3K,L). We identified at least two components in each of these mechanisms or pathways (Table 2), reinforcing their roles in dendritic development.

### The Chaperonin CCT Participates in Dendrite Morphogenesis

**FIGURE 4:**
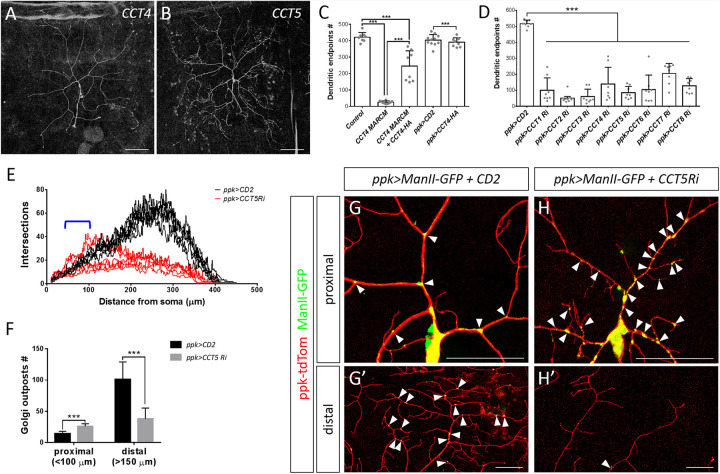
The chaperonin CCT participates in dendrite morphogenesis of c4da neurons. **(A,B)** Representative c4da MARCM clones of *CCT4*^*KG09280*^ **(A)** and *CCT5*^*k06005*^ **(B)**. Scale bars = 100 μm. **(C,D)** Quantification of numbers of dendritic endpoints in c4da neurons for the indicated genotypes. **(E)** Sholl analysis profiles for CD2 expression control and *CCT5* knockdown c4da neurons. Blue open bracket indicates the proximal regions where two *ppk>CCT5-RNAi* neurons contain more dendrites than *ppk>CD2* neurons. **(F)** Quantification of the number of Golgi outposts in proximal and distal dendrites of the dorsal region of ddaC neurons. **(G,H)** Representative c4da neurons of *ppk>CD2* **(G)** and *ppk>CCT5-RNAi* **(H)** lines. Dendritic morphologies were labeled using *ppk-tdTom* expression, and α-mannosidase II–GFP (ManII-GFP) was used as a marker for Golgi outposts. Arrowheads point to Golgi outposts in proximal regions **(G,H)** and in distal regions (**G′,H′**). Scale bars = 50 μm. Student’s *t*-test *P* values (***P < 0.001).

We chose to investigate the CCT complex further because mutants for its core subunits CCT4 and CCT5 presented strongly retarded dendritic growth (Figures 2F, 4A,B). Expression of the HA-tagged transgene *CCT4-HA* significantly restored the dendritic defect displayed by *CCT4* MARCM neurons, indicating that loss of CCT4 is indeed responsible for the aberrant dendritic morphology (Figure 4C). The partial restoration, as compared to the control, might be due to the late expression driven by *ppk-GAL4*, which failed to compensate earlier defects caused by MARCM-induced deficiency in the early embryonic stage. Alternatively, the expression level might not be sufficient for a full rescue.

To investigate if all CCT subunits are required for dendritic growth, we depleted individual CCT subunits in c4da neurons by means of RNAi. We observed reduced dendritic branching upon depletion of each CCT subunit, suggesting that CCT functions as a complex to regulate dendritic growth (Figure 4D and Supplementary Figure S2), which is supported by the fact that all subunits are assembled as a functional hetero-oligomeric complex in both *Drosophila* and mammals (Kunisawa and Shastri, 2003; Palumbo et al., 2015). We also traced axonal fascicles in *CCT5*- or *CCT1*-depleted c4da neurons and found that the pattern was indistinguishable from that of *ppk>mCD8GFP* control neurons (Supplementary Figure S3). As axonal development proceeds earlier than dendritic morphogenesis, the lack of axonal phenotypes would need further examination.

Dendritic patterning of c4da neurons begins from late embryonic stages and then undergoes scaled growth to cover the entire arborization field by the third instar larval stage (Jan and Jan, 2003). Compared with control c4da neurons, we found that the number of dendrites in *CCT5* knockdown neurons was only slightly diminished 72 h after egg laying (AEL). Strikingly, dendritic branching of *CCT5-RNAi* neurons seemed to cease at this time-point, as the number of branches at 120 h AEL was comparable to that at 72 h AEL (Supplementary Figure S4A). However, the lower-order trunks of dendrites in *CCT5-RNAi* neurons extended as much as those of control neurons (Supplementary Figure S4B). The data suggest that higher-order dendritic branching might be more sensitive to CCT-mediated protein folding. It is possible that the residual CCT5 protein level in *CCT5-RNAi* neurons is sufficient to support the growth of lower-order dendrites. Alternatively, CCT5 might be relatively specific for higher-order branches such as by a regulation of higher-order-specific substrates by the CCT chaperonin. Apart from the reduced number of branches displayed by *CCT5-RNAi* neurons, we noticed that remaining branches in *CCT5-RNAi* neurons were highly variable in their distribution along the proximodistal axis, as shown by Sholl analysis (Figure 4E), raising the possibility that the phenotype might be caused by a defect in dendritic trafficking. We examined the distribution of the Golgi outposts that are transported by dynein motors to distal regions to regulate branching (Horton et al., 2005; Ye et al., 2007; Lin et al., 2015). Numbers of Golgi outposts were increased in the proximal but reduced in the distal segments of *CCT5-RNAi* neurons (Figure 4F–H), supporting the differential branching pattern of *CCT5-RNAi* neurons along the proximodistal axis.

Using available CCT1 antibodies for immunofluorescence staining, we observed relatively equal CCT1 signal intensities in the cytoplasm of different types of da neurons (Supplementary Figure S4C). Therefore, we examined if loss of CCT subunits affects c1da neurons, which have simple comb-like arbors. Compared with control neurons, c1da neurons in which *CCT4* or *CCT5* were downregulated exhibited fewer dendritic branches (Supplementary Figures S4D–G), indicating that da neurons with either simple or complex dendritic arbors require CCT for dendritic branching.

### CCT Subunits Localize to Dendrites and Regulate Dendritic Microtubule Biogenesis

**FIGURE 5:**
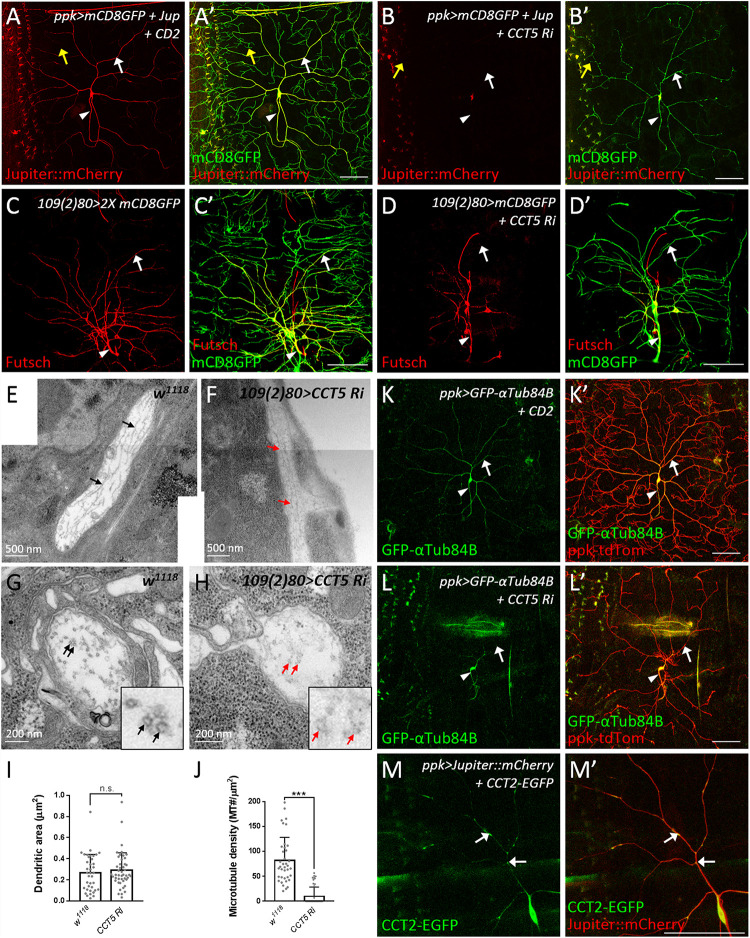
CCT regulates microtubule levels. **(A,B)** Representative images of CD2 expression control **(A)** or *CCT5* knockdown **(B)** c4da neurons exhibiting *mCD8::GFP* (green; marker for dendrites) and *Jupiter::mCherry* (red; marker for microtubules) fluorescence labeling. White arrows indicate dendritic trunks. Yellow arrows indicate terminal branches. Arrowheads indicate axons. **(C,D)** Representative images of *mCD8::GFP* (green) and Futsch immunoreactivity (red) in all da neurons of control **(C)** and *CCT5* knockdown **(D)** lines. Arrows indicate dendritic trunks. Arrowheads indicate the axons. **(E,F)** Representative figures assembledfrom several electron micrographs showing longitudinal sections of dendrites from da neurons in third instar larvae of *w*^*1118*^ **(E)** and *CCT5-RNAi* **(F)**. Arrows point microtubules. **(G,H)** Representative electron micrographs showing transverse sections of dendrites from da neurons in third instar larvae. Circular transverse microtubule profiles of approximately 25 nm in size were observed (black arrows). Clusters of microtubule cross-sections are observable in *w*^1118^ (**G**, black arrows), whereas *CCT5-RNAi* neurons exhibited fewer and non-clustered microtubule cross-sections (**H**, red arrows). Magnified views of the indicated regions were shown. **(I)** Quantification of dendritic cross-section areas in *w*^1118^ and *CCT5-RNAi* neurons. **(J)** Quantification of microtubule density in *w*^1118^ and *CCT5-RNAi* dendrites. **(K,L)** Representative images of c4da neurons displaying *ppk-tdTom* (red) and *GFP-αTub84B* (green) fluorescence labeling in control **(K)** and *CCT5-RNAi* **(L)** lines. Arrows indicates dendritic trunks. Arrowheads indicate the axons. **(M)** Representative images of c4da neurons displaying *Jupiter::mCherry* (red) and CCT2-EGFP (green, arrows) fluorescence signal. Scale bar = 100 μm. Student’s *t*-test *P* values (***P < 0.001).

Microtubules are polymers composed of α- and β-tubulin that provide the structural basis for lower-order dendrites and serve as tracks for intracellular transport of cargos including Golgi outposts (Satoh et al., 2008; Zheng et al., 2008; Ori-McKenney et al., 2012). We monitored microtubules by the microtubule-associated protein mCherry::Jupiter (Cabernard and Doe, 2009) and found that the signals were enriched in the cell body, the axon, and lower-order dendrites (Figure 5A), but low or almost undetectable in higher-order and terminal dendritic branches (yellow arrows in Figure 5A). However, upon RNAi-mediated *CCT5* depletion, the *mCherry::Jupiter* signals in dendrites and the axon became barely detectable and signal intensity in the cell body, although still detectable, was greatly reduced (Figure 5B). We also examined stabilized microtubules by performing immunostaining for the microtubule-associated protein Futsch, the *Drosophila* ortholog of MAP1B in human (Hummel et al., 2000; Roos et al., 2000; Jinushi-Nakao et al., 2007). Depletion of *CCT5* dramatically reduced the levels of stabilized microtubules in all classes of da neurons (Figures 5C,D). We further assessed the ultrastructural organization of microtubules by transmission electron microscopy (TEM). We recognized dendrites that locate between epidermal cells and the extracellular matrix in the dorsal field (Yang et al., 2019). In longitudinal sections, wild-type microtubules are aligned and cross-connected (*n* = 38) (Figure 5E). However, the microtubules were sparsely distributed and separated in *CCT5-RNAi* sections; only 23.9% *CCT5-RNAi* sections (*n* = 46) exhibited wild-type morphology of microtubules (Figure 5F). The microtubule density was significant reduced in *CCT5-RNAi* dendrites relative to control upon examining comparably sized dendritic cross-sections (Figures 5G–J). Thus, the lack of CCT5 caused a severe reduction and disorganization of microtubules in c4da dendrites.

CCT-mediated protein folding activity is required for microtubule polymerization (Yaffe et al., 1992). We investigated how absence of CCT activity might impact α-tubulin levels in c4da neurons overexpressing GFP-αTub84B (Grieder et al., 2000). α-Tubulin was observed in the cell body, axon, and lower-order dendrites of control neurons. RNAi-mediated depletion of *CCT5* caused a severe reduction in GFP-αTub84B signal in dendrites (Figures 5K,L), suggesting that lower tubulin levels might cause microtubule deficiency and reduced branching in *CCT5-*depleted neurons. To confirm this notion, we depleted c4da neurons of α- or β-tubulin and found that, in both cases, microtubule signal and dendritic branching were reduced (Supplementary Figures S5A–D). These results support the idea that the CCT complex is required for proper folding of tubulin subunits and, consequently, microtubule growth, a critical step in dendritic branching. Given that a severe reduction in tubulin stability and polymerization has been observed previously in *CCT1*-depleted larvae (Palumbo et al., 2015), we propose that unfolded tubulins not incorporated into microtubules are degraded, resulting in a microtubule deficiency in dendrites.

To understand if CCT functions in c4da dendrites, we examined the localization of CCT2-EGFP. CCT2-EGFP signal was detected in the cell body, axon, and dendrites of c4da neurons (Figure 5M). Interestingly, dendritic CCT2-EGFP signal was confined to *mCherry::Jupiter*-positive lower-order dendrites, with some signal appearing punctate and localized at branch points and shafts (arrows in Figure 5M). We also detected localization of CCT4-HA in lower-order dendrites (Supplementary Figure S5E). These dendritic signals suggest that tubulin subunits might be folded locally by CCT for microtubule polymerization during dendrite growth. Microtubules provide structural support and serve as tracks for transport of Golgi outposts (Ye et al., 2007; Lin et al., 2015). On the other hand, Golgi outposts act as microtubule nucleation center for dendrite branching (Ori-McKenney et al., 2012). Thus, CCT-mediated tubulin folding might regulate microtubule homeostasis in dendrites, which is important for transport of organelles such as Golgi outposts to shape dendritic morphology.

Actin, another critical constituent of dendrites, is a CCT client. Depletion of *CCT5* from c4da neurons did not noticeably alter Lifeact-GFP-labeled actin filaments in lower-order dendrites or in the remaining terminal branches (Supplementary Figure S6). Consistent with this finding, loss of CCT did not affect actin level or polymerization in whole larval lysates in a previous study (Palumbo et al., 2015), and *CCT2* depletion was shown not to reduce *GMA::GFP*-labeled actin filaments in lower-order dendrites (Das et al., 2017). Together, these observations indicate that actin might not be compromised directly, or as severely as microtubules, in the dendrites of CCT-depleted neurons.

Translation initiation factors eIF3b, eIF3i, and eIF3h are thought to be clients of CCT for protein folding (Olesnicky et al., 2014; Roobol et al., 2014). We also identified eIF3h as a regulator of dendrite development (Table 1), although how CCT interacts with eIF3h in dendrites awaits further study. Thus, apart from tubulins, clients of CCT are involved in diverse mechanisms/pathways that might contribute to CCT-regulated dendrite morphogenesis.

Mutations of *CCT4* and *CCT5* have been linked to the rare group of disorders hereditary sensory neuropathy (HSN), characterized by degeneration of the nerve fibers in peripheral sensory neurons and frequent progression of painless injuries (Lee et al., 2003; Bouhouche et al., 2006; Auer-Grumbach, 2013). However, the molecular basis underlying HSN is poorly understood. The *Drosophila* c4da neuron functions in nociception, and reduced dendritic complexity has been correlated with reduced nociceptive responses (Hwang et al., 2007; Ferreira et al., 2014; Honjo et al., 2016). Hence, depletion of CCT might mimic symptoms of HSN patients, and thus could be used as a *Drosophila* model of HSN to unravel the molecular basis of this rare disorder and contributory pathogenic networks.

### Neurological Disorders Are Associated With Protein Folding Machineries

Our screen also identified two chaperones, Hsc70-5 and Hsp83 (Figures 2F, 3M,N), indicating that protein folding machineries are required for dendritic development. Protein misfolding is a critical factor in the pathogenesis of several neurodegenerative diseases (Valastyan and Lindquist, 2014). Indeed, decreased levels of the human chaperone Mortalin are associated with Alzheimer’s disease (AD) and Parkinson’s diseases (PD) (De Mena et al., 2009; Qu et al., 2012). Similarly, knockdown of the *Drosophila* Mortalin ortholog, Hsc70-5, causes loss of synaptic mitochondria in a *Drosophila* PD model (Zhu et al., 2013). Highly abundant HSP90 is the human ortholog of *Drosophila* Hsp83 and it mediates many basic cellular processes (Li et al., 2012). It has previously been shown that mRNA levels of HSP90 and CCT subunits are repressed in AD patients (Brehme et al., 2014). Furthermore, both HSP90 and CCT subunits interact with Huntingtin, and respective loss-of-function mutations modify neuronal dysfunction in a *Drosophila* model for Huntington’s disease (Shirasaki et al., 2012). Further study of protein folding machineries and the interaction networks of their clients in *Drosophila* dendrites will contribute to a better understanding of the pathogenesis of these and other neurological disorders.

### Other Identified Mechanisms and Pathways

#### Protein Phosphatase Type 2A (PP2A) Holoenzyme

**FIGURE 6:**
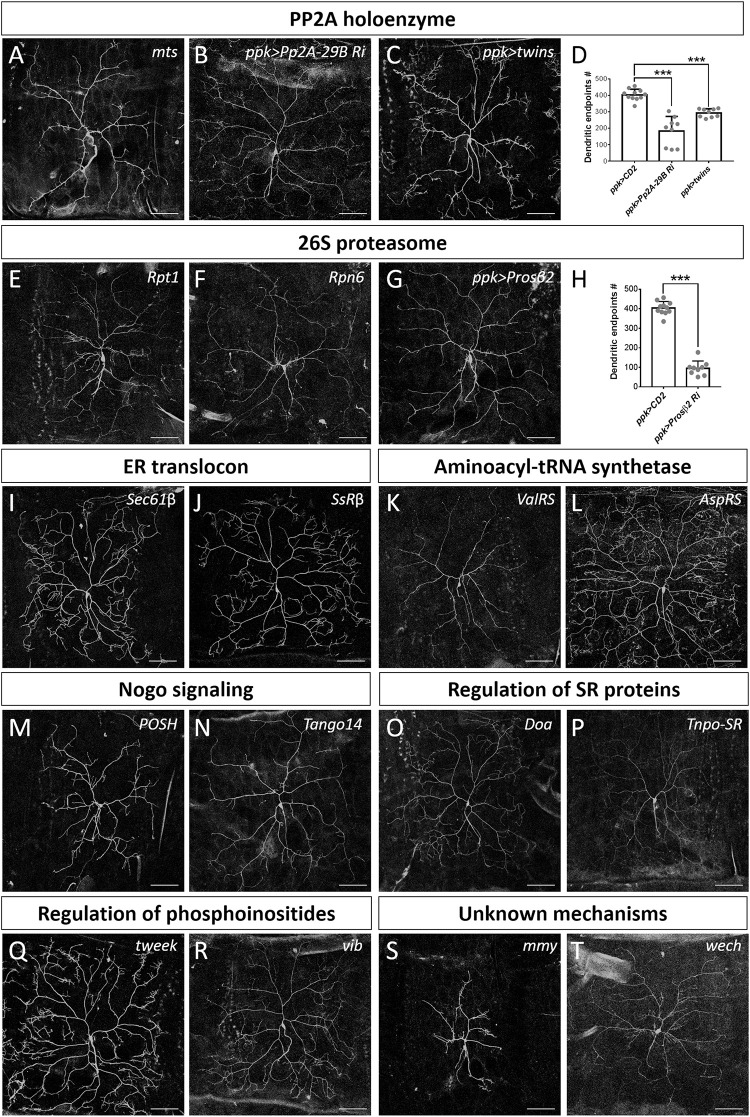
Dendrite regulators acting in conserved complexes or pathways. **(A–D)** Components of PP2A holoenzyme are required for dendrite morphogenesis. Dendritic arborization of *mts*^*s5286*^ c4da MARCM clones is shown **(A)**. Dendritic arborization of *Pp2A-29B-RNAi* **(B)** and *twins-*overexpressing **(C)** c4da neurons was visualized with *mCD8::GFP* driven by *ppk-GAL4*. Quantification of numbers of dendritic endpoints in c4da neurons of the indicated genotypes is shown in **(D)**. **(E–H)** The 26S proteasome is required for dendrite morphogenesis. Dendritic arborization of *Rpt1*^*k11110*^ **(E)** and *Rpn6*^*k00103*^ **(F)** c4da MARCM clones is shown. Dendritic arborization of *Prosβ2-RNAi* c4da neurons was visualized with *mCD8::GFP* driven by *ppk-GAL4* **(G)**, and dendritic endpoints were enumerated **(H)**. **(I–T)** Representative c4da MARCM clones of indicated mutants. Alleles used were *Sec61β^*k03307*^* **(I)**, *SsRβ^*s1939*^* **(J)**, *ValRS*^*k14804*^ **(K)**, *AspRS*^*KG03912*^ **(L)**, *POSH*^*k15815*^ **(M)**, *Tango14*^*k00619*^ **(N)**, *Doa*^*s2784*^ **(O)** *Tnpo-SR^*KG04870*^* **(P)**, *tweek*^*EY20585*^ **(Q)**, *vib*^*j7A3*^ **(R)**, *mmy*^*KG08617*^ **(S)**, and *wech*^*k08815*^ **(T)**. Scale bars = 100 μm. Student’s *t*-test *P* values (***P < 0.001).

The PP2A complex is composed of the scaffold subunit PP2A-A, the regulatory subunit PP2A-B, and the catalytic subunit PP2A-C (Shi, 2009). Our screen identified that mutation of the *Drosophila* ortholog of the catalytic subunit, Mts, induced a reduction in dendritic branching (Figures 2F, 6A). To assess if PP2A in *Drosophila* is required as a complex in dendritic development, we examined the requirement for *PP2A-29B* (which encode PP2A-A) by RNAi knockdown approach. Numbers of dendritic branches were reduced and terminal branches were shortened in *PP2A-29B-RNAi* c4da neurons (Figures 6B,D), recapitulating the phenotypes observed in *mts* clones. Given the similarities in phenotypes induced by mutations of individual PP2A subunits, PP2A appears to function as a complex in dendritic development. The stoichiometries of the component subunits of a functional complex are typically tightly controlled, and overexpression of individual subunits could lead to loss-of-function phenotypes. Indeed, overexpression of the regulatory subunit encoded by *twins* has been shown to reduce PP2A activity (Wang et al., 2011), and overexpressing *twins* induced a reduction of dendritic branching (Figures 6C,D). Similarly, overexpression of *ppp2r2b*, the human ortholog of *twins*, suppresses dendritic outgrowth in rat hippocampal neurons, evidencing functional conservation of how the PP2A complex regulates dendritic morphology (Dickey and Strack, 2011) and demonstrating a crucial role for the PP2A holoenzyme in dendritic development. The finding in dendritic growth is consistent with recent reports that also suggest the involvement of the PP2A complex in dendrite pruning (Rui et al., 2020; Wolterhoff et al., 2020). The PP2A complex is known to be involved in various signaling pathways to regulate diverse cellular processes (Casso et al., 2008; Shi, 2009; Zhang et al., 2009). It would be interesting to study PP2A-regulated signaling pathways in dendrite development and pruning.

#### The 26S Proteasome

The 26S proteasome is composed of the 19S regulatory particle (in which the ATPase subunit Rpt1 and the non-ATPase subunit Rpn6 interact; Guruharsha et al., 2011; Bar-Nun and Glickman, 2012) and the 20S core particle. We identified that loss of Rpt1 or Rpn6 activity in the MARCM clones reduced dendritic arborization (Figures 2F, 6E,F), suggesting that the 19S particle is involved in dendritic development. To test if the 20S core particle also contributes to dendrite morphogenesis, we depleted its catalytic β*2* subunit by means of *Prosβ2* RNAi knockdown and observed a concomitant reduction in dendritic branching (Figures 6G,H), suggesting that the 20S core particle and the 19S regulatory particle function in concert to mediate dendrite morphogenesis. Subunits of both particles are also required for developmental pruning of c4da dendrites in the pupal stage (Kuo et al., 2005; Rumpf et al., 2014). Thus, protein degradation by the 26S proteasome is critical in two distinct stages of dendritic development.

#### The Endoplasmic Reticulum (ER) Translocon

Co-translational translocation of proteins across the ER membrane requires the Sec61 translocon and the translocon-associated protein (TRAP) complex (Hartmann et al., 1993, 1994). We found that MARCM mutant neurons for *Sec61*β as well as *SsR*β that encodes a TRAP component exhibited reduced dendritic branching, indicating that the ER translocon is required for dendrite development (Figures 2F, 6I,J). Sec61 subunits have been found to localize in dendrites and are required for growth of cultured neurons (Pierce et al., 2000; Aridor et al., 2004; Sepp et al., 2008). Together, these data suggest that the ER translocon might locally coordinate protein translation and translocation during dendritic growth and branching.

#### Aminoacyl-tRNA Synthetases (aaRSs)

aaRSs ligate amino acids to their cognate tRNAs, allowing these aminoacyl-tRNAs to be used for nascent polypeptide synthesis (Lu et al., 2015). Mutants for *Glycyl-tRNA synthetase*, *Tryptophanyl-tRNA synthetase*, or *Glutaminyl-tRNA synthetase* exhibit reduced dendrites in *Drosophila* olfactory projection neurons (Chihara et al., 2007). Our screen identified Valyl-tRNA synthetase as a positive regulator of dendrites (Figures 2F, 6K). To test if other aaRSs are also involved in dendritic regulation, we examined the contribution of *Aspartyl-tRNA synthetase (AspRS)* to dendrite growth using a respective P-insertion mutant and RNAi knockdown. Surprisingly, we observed normal dendritic morphology in both *AspRS* mutant and *AspRS-RNAi* neurons (Figure 6L and Supplementary Table S3), indicating that aaRSs may exert individual effects in c4da dendrite development. Further investigation is necessary to elucidate the roles of all aaRSs in dendrite morphogenesis.

#### Nogo Signaling

Three mammalian Nogo isoforms, i.e., Nogo-A, Nogo-B and Nogo-C, interact with respective receptors to activate downstream effectors and regulate gene expression and microtubule stabilization (Schwab, 2010; Sui et al., 2015). We identified two components of Nogo signaling, POSH and Tango14, as being dendrite regulators (Figures 2F, 6M,N). POSH acts downstream of the Nogo-A receptor to inhibit axon outgrowth in mammalian neurons (Dickson et al., 2010). Tango14 is orthologous to the Nogo-B receptor (NgBR) required for axonal branching and extension in sensory neurons (Eckharter et al., 2015). Nogo-A expression is required for proper dendritic complexity and length in mice (Petrinovic et al., 2013; Zemmar et al., 2018), and aberrant Nogo-A signaling has been implicated in several neurodegenerative diseases such as AD, amyotrophic lateral sclerosis, and multiple sclerosis (Schmandke et al., 2014). A loss-of-function mutation in NgBR causes a congenital glycosylation disorder associated with severe neurological impairments (Park et al., 2014). Our study evidences a conserved role of Nogo signaling in *Drosophila* dendritic development, necessitating further investigation of the two predicted Nogo genes in *Drosophila* (Schwab, 2010).

#### Serine-Arginine-Rich (SR) Proteins

SR proteins belong to a conserved splicing factor family essential for RNA splicing and other post-transcriptional modifications (Bradley et al., 2015; Howard and Sanford, 2015). SR proteins are phosphorylated by the protein kinase Doa, and they require Tnpo-SR for nuclear import (Du et al., 1998; Allemand et al., 2002). We show from our screen that both Doa and Tnpo-SR are required for dendritic arborization (Figures 2F, 6O,P). The SR protein X16, which is required for proper dendritic morphology in c4da neurons, is regulated by Doa and Tnpo-SR (Allemand et al., 2002; Wan et al., 2008; Olesnicky et al., 2014). Thus, Doa and Tnpo-SR potentially regulate the function of X16 in mediating c4da dendritic morphology.

#### Regulation of Phosphoinositides

Phosphoinositides are phosphorylated derivatives of phosphatidylinositol (PI) and they participate in a vast array of biological processes, including vesicle trafficking and actin dynamics (Balla, 2013). Although PIs have been reported previously to contribute to dendritic branching of cultured neurons (Li et al., 2008; Zhang et al., 2017), their roles remain poorly understood. Our screen revealed two regulators of phosphoinositides, Tweek and Vib, as controlling dendritic development (Figures 2F, 6Q,R). Tweek is known to regulate the level of PI(4,5)P_2_ at synapses (Verstreken et al., 2009), whereas Vib has been implicated in neuroblast development and its human orthologs are class I PI transfer proteins, PITPα and PITPβ (Gatt and Glover, 2006; Giansanti et al., 2006; Cockcroft and Garner, 2013). PITPα localizes in dendrites and plays a role in neurite growth (Xie et al., 2005; Cosker et al., 2008). Our identification of Tweek and Vib support that PIs participate in c4da dendrite development, further expanding our understanding of their conserved roles and regulatory mechanisms (Balakrishnan et al., 2015).

#### Mmy and Wech

Mmy is a conserved enzyme that catalyzes the formation of UDP-*N*-acetylglucosamine, which is needed for *N*- and *O*-linked protein glycosylation and biosynthesis of GPI anchors (Araujo et al., 2005; Schimmelpfeng et al., 2006; Tonning et al., 2006). We observed that *mmy* mutant neurons exhibited severely constrained dendritic branching (Figure 2F, 6S). Mmy is a known antagonist of Dpp signaling (Humphreys et al., 2013). However, perturbing Dpp signaling in c4da neurons was previously shown to only induce mild dendritic defects (Follansbee et al., 2017), suggesting that Mmy-mediated regulation of dendritic morphology is Dpp-independent.

Wech is crucial for integrin–cytoskeleton linkage (Loer et al., 2008). Integrin depletion mildly impacts dendrite branching, primarily due to detachment from the extracellular matrix (Han et al., 2012; Kim et al., 2012). Surprisingly, we did not observe an anticipated detachment phenotype in our c4da MARCM clones upon Wech depletion (Figures 2F, 6T). Instead, we observed that dendritic branch number declined severely and *Venus::pm* fluorescence signal became fainter (Figure 2F), suggesting that Wech exerts integrin-independent regulation of dendrites. Further studies of Mmy and Wech are needed to characterize the mechanisms by which they regulate dendritic development.

## Conclusion

Previous EMS-based screens by others have generated less than 1% mutants bearing dendritic defects (Gao et al., 1999; Ye et al., 2007). In contrast, our screen revealed abnormal dendritic morphology in 19% (52/280) of the P-insertion mutants, indicating high efficiency in identifying regulators of dendritic branching. The collection of P-insertion mutants we screened all perturb eye development (Chen et al., 2005; Call et al., 2007), with the respective impacted proteins representing key components of major developmental and cellular pathways. Therefore, it is perhaps not surprising that we uncovered a much higher percentage of mutants in our screen. Our analysis indicates that the identified genes and mechanisms are commonly involved in the development of both c4da dendrites and the compound eye, likely at cellular levels. Since the compound eye is an easier system for phenotypic observation, pilot screens using eyes could be deployed to establish the crucial components of major mechanisms and novel pathways, thereby facilitating more intensive dendrite morphological screens for validation. In conclusion, our strategy of using high-efficiency SOP-FLP-based MARCM clone generation to target a collection of pre-screened P-insertion mutants serves as a foundation to illustrate the overall blueprint of dendrite generation during development.

## Materials and Methods

### SOP-FLP-Based MARCM Screen

We individually crossed 280 autosomal P-insertion strains exhibiting eye defects in clonal analyses (Chen et al., 2005) to the following SOP-FLP-based MARCM-ready flies: *GAL4^5–40^ UAS-Venus::pm SOP-FLP^#42^; Tub-GAL80 FRT40A/CyO* (DGRC# 109947); *GAL4^5–40^ UAS-Venus::pm SOP-FLP^#42^; FRT42D Tub-GAL80/CyO* (DGRC# 109949); *GAL4^5–40^ UAS-Venus::pm SOP-FLP^#42^; FRT82B Tub-GAL80/TM6B* (DGRC# 109951); and *GAL4^5–40^ UAS-Venus::pm SOP-FLP^#42^; Tub-GAL80 FRT80B/TM6B* (a derivative of DGRC# 109951). *FRT40A* was crossed to *GAL4^5–40^ UAS-Venus::pm SOP-FLP^#42^; Tub-GAL80 FRT40A/CyO* as a control for MARCM clones. We examined dendritic morphologies of MARCM clones at the wandering larval stage incubated at 25°C. The fly strains for our SOP-FLP-based MARCM screen and for validation tests are listed in Supplementary Table S1–S3.

### Fly Stocks

Fly stocks were obtained from the Bloomington Drosophila Stock Center (BDSC), the Drosophila Genetic Resource Center (DGRC), and the Vienna Drosophila Resource Center (VDRC). GAL4 drivers were *ppk-GAL4* (Kuo et al., 2005) and *109(2)80-GAL4* (Gao et al., 1999). UAS transgenic stocks were *UAS-mCD8-GFP* (Gao et al., 1999), *UAS-CD2* (BDSC# 1373), *UAS-ManII-GFP* (Ye et al., 2007), *UAS-Jupiter::mCherry* (Cabernard and Doe, 2009), *UAS-GFP-αTub84B* (Grieder et al., 2000), *UAS-CCT2-EGFP* (BDSC# 53755), *UAS-twins* (Bajpai et al., 2004), and *UAS-Lifeact-GFP* (Hatan et al., 2011). *UAS-CCT4-HA* was a fusion of C-tagged HA to CCT4 cDNA (RE61939, DGRC) in pUAST. We obtained *ppk-CD4-tdTomato* (Han et al., 2011) for labeling c4da neurons from BDSC. RNAi knockdown strains were *UAS-CCT1 RNAi* (BDSC# 32854), *UAS-CCT2 RNAi* (BDSC# 34711), *UAS-CCT3 RNAi* (BDSC# 34969), *UAS-CCT4 RNAi* (VDRC# 22154), *UAS-CCT5 RNAi* (BDSC# 41818), *UAS-CCT6 RNAi* (BDSC#43146), *UAS-CCT7 RNAi* (BDSC# 34931), *UAS-CCT8 RNAi* (VDRC# 103905), *UAS-αTub84B RNAi* (VDRC# 33427), *UAS-βTub60D RNAi* (VDRC# 34606), *UAS-Pp2A-29B RNAi* (BDSC# 50533), and *UAS-Prosβ2RNAi* (VDRC# 103575). We examined the dendritic morphologies of *RNAi* lines for knockdown of all CCT components and for α- and β-tubulin subunits at the wandering larval stage incubated at 29°C.

### Image Collection, Quantification, and Statistical Analysis

The c4da neurons from the second through sixth abdominal segment were imaged on a Zeiss LSM510 microscope. Total dendritic endpoints for 6–13 neurons of each genotype were manually counted using the Cell Counter ImageJ plugin to quantify dendritic branching. To quantify Golgi outposts, ManII-GFP puncta in proximal (<100 μm from the soma) and distal (>150 μm from the soma) dendrites in dorsal regions of ddaC neurons were counted using the Cell Counter ImageJ plugin. To obtain dendrite features enabling quantification of total dendrite length and profile, dendrites were traced using the NeuronJ ImageJ plugin. The Sholl Analysis Plugin of ImageJ was used to analyze dendrite profiles. A series of concentric circles at 1 μm intervals and centered on the cell body were generated, and the number of dendrites intersecting each circle was then calculated. Pairwise comparisons of group means were performed by Student’s *t*-test. All statistical analyses were performed using Prism 7.0 (GraphPad Software). Data are shown as the mean ± SD, with number of asterisks indicating the significance of *P* values (^∗^*P* < 0.05; ^∗∗^*P* < 0.01; ^∗∗∗^*P* < 0.001).

### Immunohistochemistry and TEM

The wandering larvae were dissected and prepared for immunohistochemistry as described previously (Shrestha and Grueber, 2011). Primary antibodies used in immunostaining included rat anti-CCT1 (1:50, Abcam), mouse anti-Futsch (1:200, Developmental Studies Hybridoma Bank), rabbit anti-GFP-Alexa488 (1:500, Life Technologies), and rabbit anti-HA (1:400; Cell Signaling Technology). Goat anti-rat Cy3 and anti-mouse Cy3 secondary antibodies were obtained from Jackson ImmunoResearch Laboratories Inc. Wandering larvae were dissected and prepared for TEM experiments as described previously (Yang et al., 2019).

## Data Availability Statement

All datasets presented inthis study are included in the article/Supplementary Material.

## Author Contributions

Y-HW performed most of the experiments and analyzed the data. Z-YD assisted in assessing Golgi outpost distribution. Y-JC performed the TEM experiments. Y-HW, M-LH, and C-TC designed and interpreted the experiments and wrote the manuscript. M-LH and C-TC supervised the project. All authors contributed to the article and approved the submitted version.

## Conflict of Interest

The authors declare that the research was conducted in the absence of any commercial or financial relationships that could be construed as a potential conflict of interest.

## Footnotes

1 http://www.bruinfly.ucla.edu/
