# Supplementary material for: An Efficient Screen for Cell-Intrinsic Factors Identifies the Chaperonin CCT and Multiple Conserved Mechanisms as Mediating Dendrite Morphogenesis
Source: Front Cell Neurosci. 2020 Sep 25;14:577315. doi: 10.3389/fncel.2020.577315 (PMC7546278; doi:10.3389/fncel.2020.577315)
Supplement: Supplementary file 1 [file Table_1.docx]

**SUPPLEMENTARY MATERIAL**


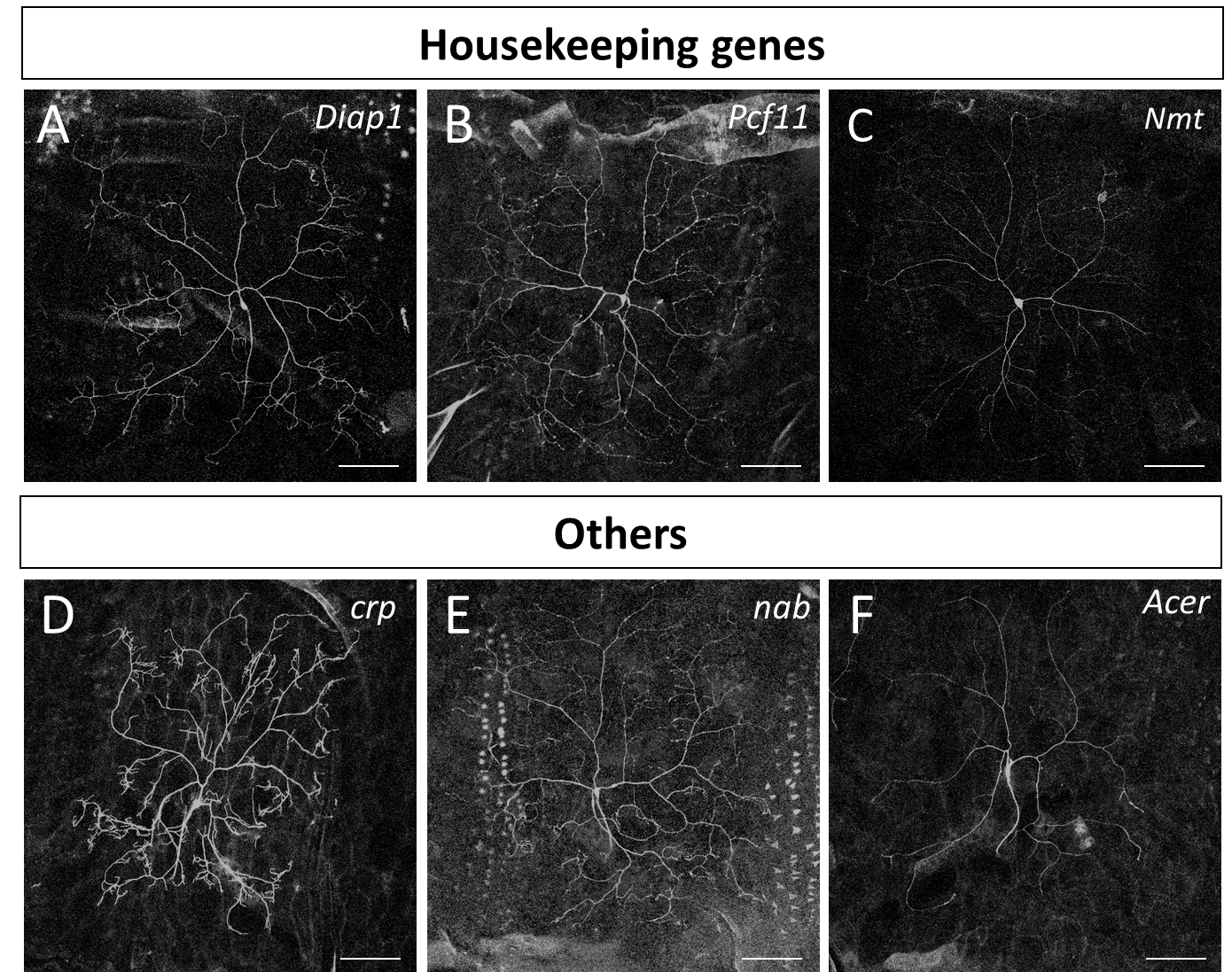


**Figure S1. Housekeeping genes and other dendrite regulators that could not be categorized. (A-F)** Representative c4da MARCM clones of indicated mutants. Alleles used were *Diap1^j5C8^* (A), *Pcf11^k08015^* (B), *Nmt^j1C7^* (C), *crp^k00809^* (D), *nab^KG07676^* (E), and *Acer^k07704^* (F). Scale bars = 100 μm.


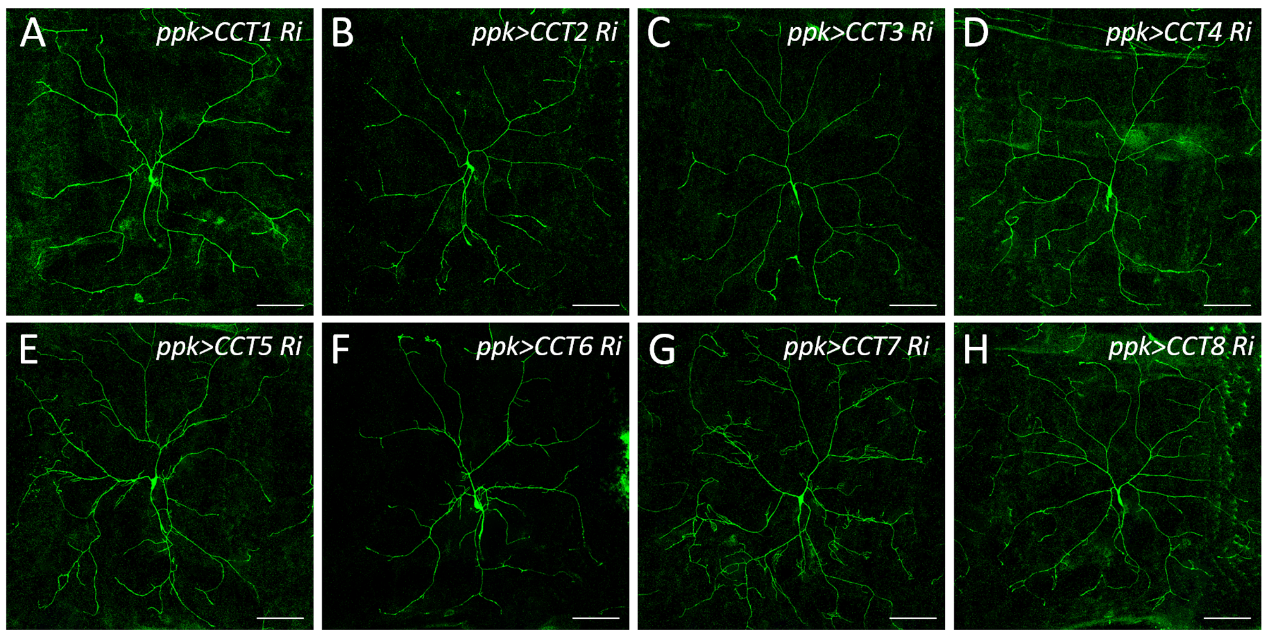


**Figure S2.** **Depletion of each CCT subunit by RNAi reduces dendritic branching. (A-H)** Representative images of c4da neurons of the indicated genotypes labeled by *mCD8::GFP* (green). Scale bars = 100 μm.

**
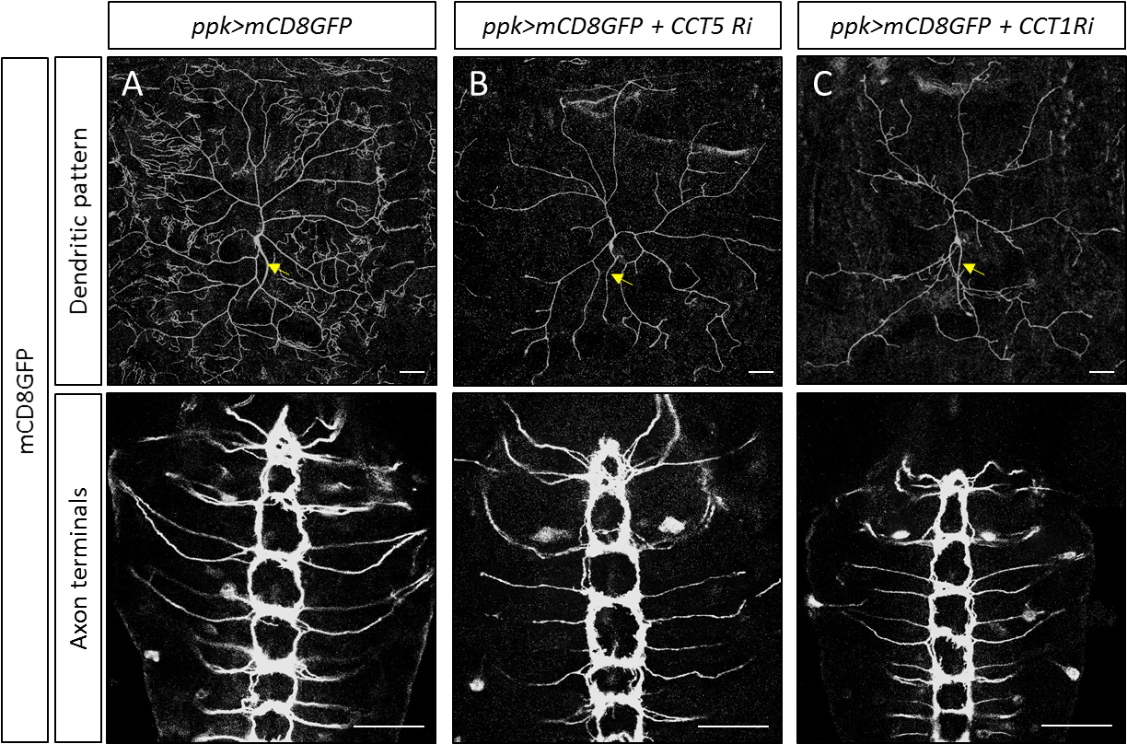
**

**Figure S3.** **Axon terminals of c4da neurons appear unaffected upon depletion of CCT subunits. (A-C)** Representative dendritic patterning (upper panels) and axon terminals in the ventral nerve cord (lower panels) of c4da neurons from third instar larvae. Compared with control (A), depletion of *CCT5* (B) or *CCT1* (C) severely inhibited dendritic arborization, whereas the patterns of axon terminals appeared unaffected. Arrows point to axons. Scale bars = 50 μm.


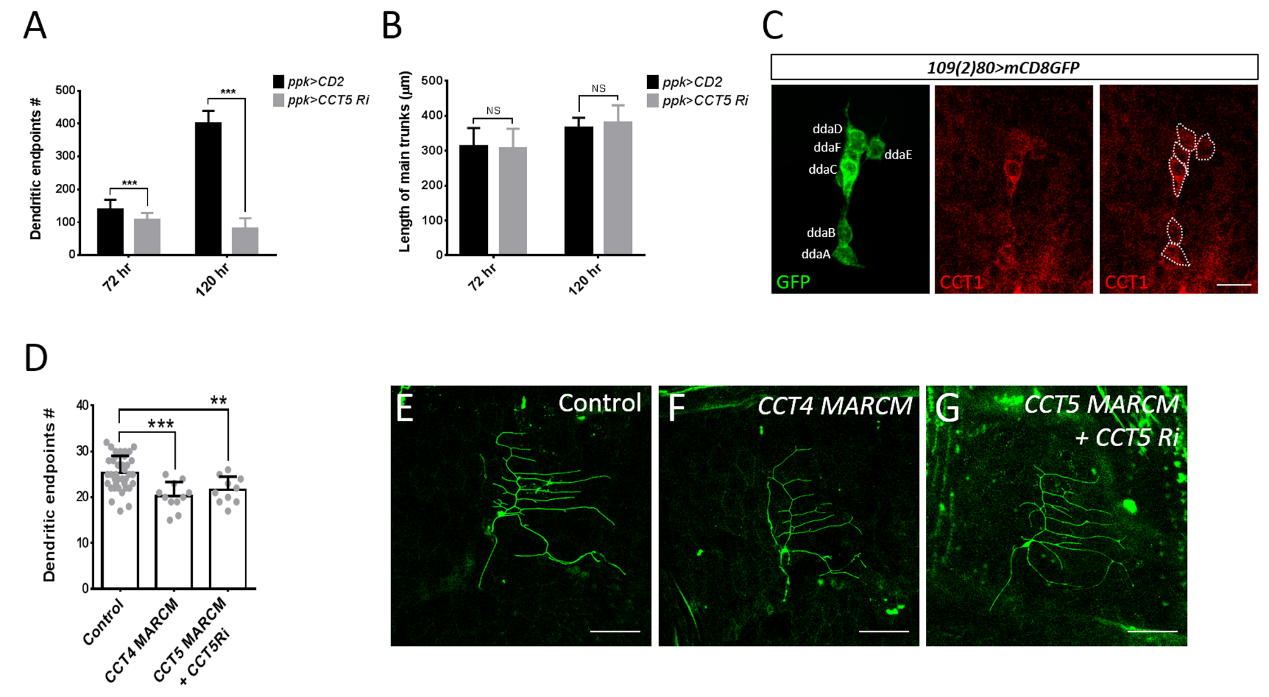


**Figure S4. CCT-mediated regulation of dendrite morphogenesis and CCT distribution in da neurons. (A)** Quantification of dendritic endpoint numbers in CD2 expression control and *CCT5* knockdown c4da neurons at 72 h or 120 h after egg laying. **(B)** Quantification of main dendritic trunk length for control and *CCT5* knockdown c4da neurons at the indicated time-points. **(C)** Visualization of *mCD8::GFP* (green) and CCT1 immunoreactivity (red) in cytoplasm of all dorsal da neurons from a stage 17 embryo: ddaD and ddaE are c1da neurons; ddaB is a c2da neuron; ddaA and ddaF are c3da neurons; ddaC is a c4da neuron. Scale bar = 10 µm. **(D)** Quantification of numbers of dendritic endpoints in c1da neurons of the indicated genotypes. **(E-G)** Representative images for c1da MARCM clones of control (E), *CCT4^KG09280^* (F), and *CCT5^k06005^* + *CCT5-RNAi* (G) neurons. Scale bars = 100 μm. Student’s t test P values (**, P < 0.01; ***, P < 0.001).

**
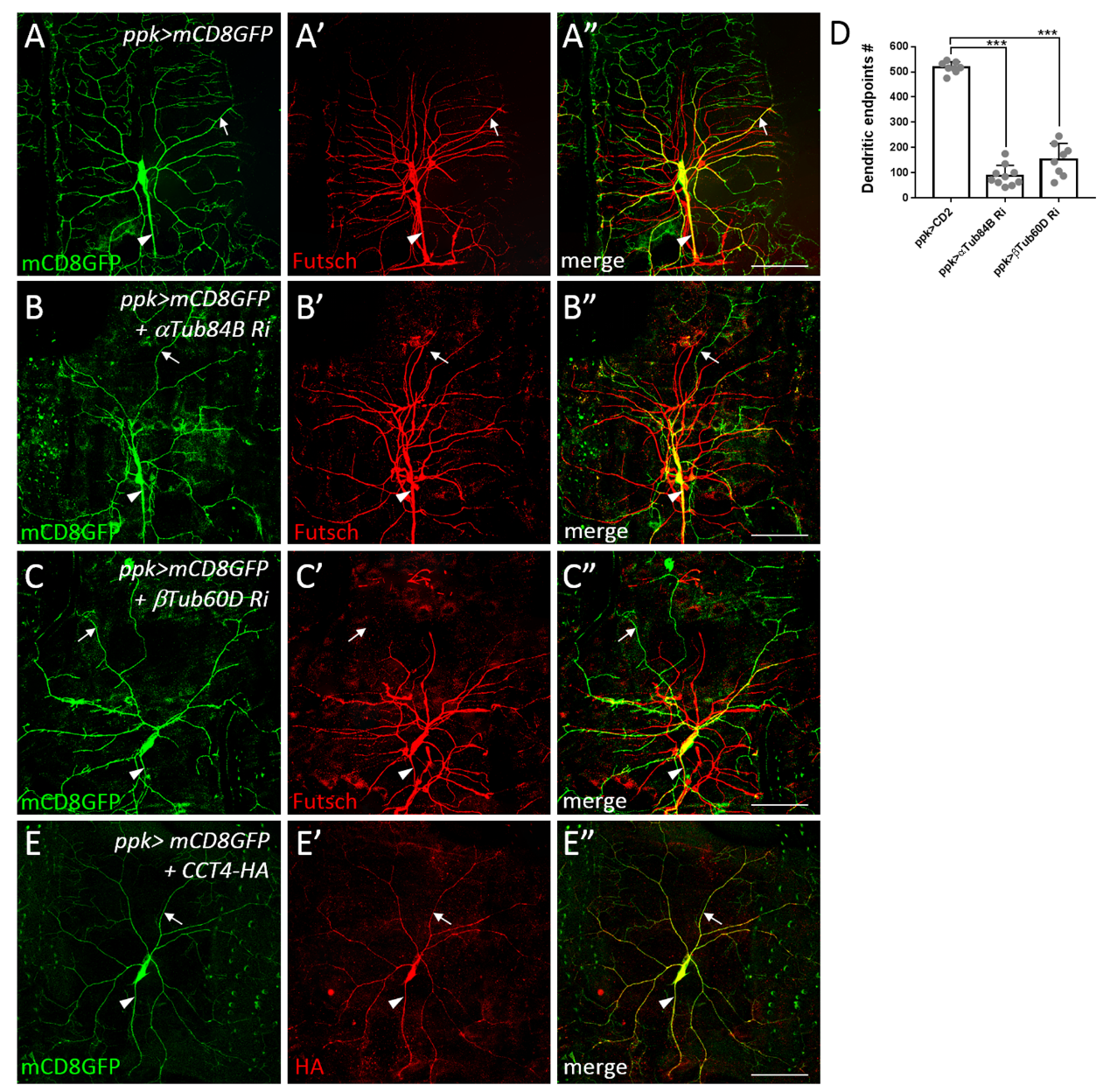
**

**Figure S5. Downregulation of tubulins phenocopies *CCT* mutants.** **(A-C)** Representative images of c4da neurons labeled by *mCD8::GFP* (green) and Futsch (red) in *ppk>mCD8GFP* control (A), or co-driving *αTub84B-RNAi* (B), or *βTub60D-RNAi* (C) lines. **(D)** Quantification of numbers of dendritic endpoints in c4da neurons of the indicated genotypes. Student’s t test P values (***, P < 0.001). **(E)** Immunostaining of CCT4-HA in *mCD8::GFP*-labeled c4da neurons by HA antibody. Arrows indicate dendritic trunks. Arrowheads indicate axons. Scale bars = 100 μm.

**
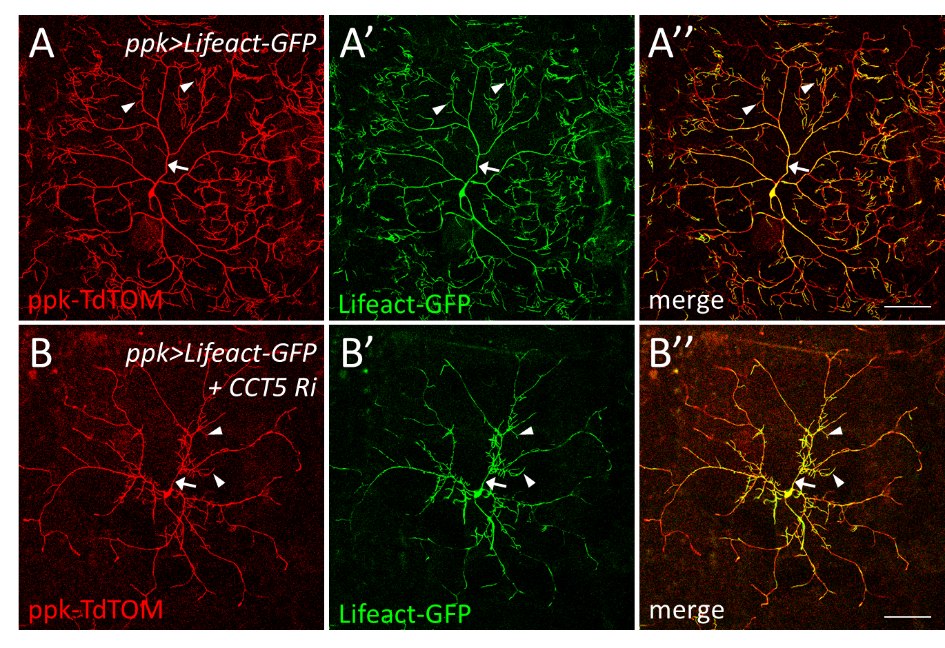
**

**Figure S6. Lifeact-GFP-labeled actin filaments are normal upon CCT5 depletion. (A-B)** Representative images of c4da neurons labeled by *ppk-TdTOM* (red) and Lifeact-GFP (green) for control (A) and *CCT5-RNAi* (B) lines. Arrows indicate dendritic trunks and arrowheads indicate terminal branches. Scale bars = 100 μm.

**Table S1. List of P-insertion strains that showed altered dendritic morphologies in our screen**

|  | **DGRC #** | **Gene** |  | **DGRC #** | **Gene** |
| --- | --- | --- | --- | --- | --- |
| 1 | 111153 | *ab* | 27 | 111676 | *nab* |
| 2 | 111221 | *Acer* | 28 | 111383 | *Nmt* |
| 3 | 111568 | *ATPsynC* | 29 | 111233 | *Pcf11* |
| 4 | 111075 | *Cam* | 30 | 111348 | *POSH* |
| 5 | 111690 | *CCT4* | 31 | 111067 | *raw* |
| 6 | 111085 | *CCT5* | 32 | 111151 | *rgr* |
| 7 | 111602 | *CG15141* | 33 | 111115 | *Rpn6* |
| 8 | 111594 | *CG42327* | 34 | 111390 | *RpS12* |
| 9 | 111604 | *CG5446* | 35 | 111139 | *RpS2* |
| 10 | 111066 | *crp* | 36 | 111098 | *Rpt1* |
| 11 | 111637 | *Cyt-c1* | 37 | 111156 | *SCAR* |
| 12 | 111353 | *DCTN2-p50* | 38 | 111073 | *Sec61β* |
| 13 | 111105 | *DCTN3-p24* | 39 | 111397 | *SsRβ* |
| 14 | 111396 | *Diap1* | 40 | 111227 | *Su(H)* |
| 15 | 111056 | *Doa* | 41 | 111188 | *Syt1* |
| 16 | 111254 | *eIF3h* | 42 | 111131 | *Tango14* |
| 17 | 111660 | *eIF5B* | 43 | 111424 | *TkR99D* |
| 18 | 111299 | *Gp150* | 44 | 111581 | *Tnpo-SR* |
| 19 | 111171 | *Hsc70-5* | 45 | 111392 | *Trl* |
| 20 | 111379 | *Hsp83* | 46 | 111414 | *trx* |
| 21 | 111093 | *lola* | 47 | 111703 | *tweek* |
| 22 | 111563 | *mmy*^a^ | 48 | 111279 | Unknow^c^ |
| 23 | 111681 | *mmy*^a^ | 49 | 111460 | Unknow^c^ |
| 24 | 111041 | *MRG15* | 50 | 111107 | *ValRS* |
| 25 | 111114 | *mts*^b^ | 51 | 111047 | *vib* |
| 26 | 111466 | *mts*^b^ | 52 | 111249 | *wech* |
| ^a^, Two allelic alleles for *mmy*. ^b^, Two allelic alleles for *mts*. ^c^, Two P-insertions are unannotated to any genes. | | | | | |

**Table S2. List of fly strains for validation experiments**

| **Gene** |  | **Methods** | **Stocks** | | |
| --- | --- | --- | --- | --- | --- |
| **DGRC #** | **Allele** | **a b c d** | **Bloomington #** | **DGRC #** | **VDRC #** |
| 111221 | *Acer^k07704^* | ■□☒□ |  |  | 3324 |
| 111568 | *ATPsynC^KG01914^* | □□■□ | 35464* |  | 106834 |
| 111075 | *Cam^k04213^* | ■■☒□ | 34609 |  | 28242, 102004 |
| 111690 | *CCT4^KG09280^* | □□■□ |  |  | 22154*, 22155*, 106099* |
| 111085 | *CCT5^k06005^* | ■■■□ | 41818* |  | 24098, 47742*, 109505* |
| 111066 | *crp^k00809^* | ■■☒■ | 31896, 37470 | 114622 | 26885, 100565 |
| 111637 | *Cyt-c1^KG05986^* | □□■□ | 34583* |  | 9180, 109809* |
| 111353 | *DCTN2-p50^k16109^* | ■■□□ |  | 103002 |  |
| 111105 | *DCTN3-p24^k14618^* | □■□□ | 12316 |  |  |
| 111396 | *Diap1^j5C8^* | ■■☒□ | 33597 | 108011 |  |
| 111056 | *Doa^s2784^* | ■■■□ | 50903 |  | 102520, 19066, 20120, 21294, 46449* |
| 111254 | *eIF3h^k09003^* | □■■□ | 55603 | 102701 | 36086*, 36087*, 106189* |
| 111660 | *eIF5B^KG09489^* | □□■□ | 44418* |  | 31365*, 31366, 109782* |
| 111299 | *Gp150^k11120b^* | ■□■□ | 32400 |  | 899*, 900, 33294, 36301, 100134 |
| 111171 | *Hsc70-5^k04907^* | □■■□ |  |  | 47745, 106236* |
| 111379 | *Hsp83^j5C2^* | ■■■□ | 32996, 33947* | 108372 | 7716*, 108568* |
| 111093 | *lola^k09901^* | ■■□□ | 10946 |  |  |
| 111681 | *mmy^KG08617^* | ■■☒■ |  | 111563 | 105829 |
| 111041 | *MRG15^j6A3^* | ■□☒□ | 35241 |  | 43800, 43802, 110618 |
| 111114 | *mts^s5286^* | ■■■■ | 27723*, 38337* | 111466, 102837, 103008 | 35171, 35172, 41924 |
| 111676 | *nab^KG07676^* | □□■□ |  |  | 6273, 24944*, 104811 |
| 111383 | *Nmt^j1C7^* | ■□■□ |  |  | 28019*, 28021 |
| 111233 | *Pcf11^k08015^* | ■■■□ | 32411* |  | 38365, 38366, 44115*, 103710 |
| 111348 | *POSH^k15815^* | ■■☒□ |  |  | 26655, 26657 |
| 111067 | *raw^k01021^* | ■■☒□ | 32393 | 102977 | 24532, 101255 |
| 111115 | *Rpn6^k00103^* | ■■■□ | 29385 |  | 18021*, 18022*, 103942* |
| 111390 | *RpS12^s2783^* | ■□■□ |  |  | 109381* |
| 111139 | *RpS2^k01215^* | □■■□ | 6262, 53319 | 101557 | 20963*, 100308* |
| 111098 | *Rpt1^k11110^* | □■■□ | 33930 |  | 17176, 47436, 50391, 108834* |
| 111073 | *Sec61β^k03307^* | □■■□ | 50626* |  | 8785, 107784* |
| 111397 | *SsRβ^s1939^* | □■☒□ |  | 107514 | 12101 |
| 111227 | *Su(H)^k07904^* | ■■☒□ | 28900 | 101292 |  |
| 111188 | *Syt1^k05909^* | □■☒□ | 3910, 4377, 31289, 31668 |  | 8874, 8875, 8876, 100608 |
| 111131 | *Tango14^k00619^* | □■☒■ | 31571 | 104590 | 42499, 108341 |
| 111581 | *Tnpo-SR^KG04870^* | □■☒□ | 7744, 25988, 56974 |  | 33569, 33571, 40991 |
| 111392 | *Trl^s2325^* | ■■☒□ | 40940, 41582, 58473 |  |  |
| 111703 | *tweek^EY02585^* | □■■■ | 39696, 39697 |  | 19306*, 26645, 102639*, 110686* |
| 111107 | *ValRS^k14804^* | □■■□ | 34338* |  | 109627*, 21782 |
| 111047 | *vib^j7A3^* | ■□□□ |  |  |  |
| 111249 | *wech^k08815^* | ■□☒□ |  |  | 41623, 106390 |
| 111153 | *ab^k02807^* | ■□☒□ | 29407 |  |  |
| 111151 | *rgr^k02605^* | □☒☒□ | 31008, 19704 |  | 6289 |
| 111156 | *SCAR^k03107^* | □☒☒□ | 8754, 31126, 51803, 36121 | | 21908 |
| 111414 | *trx^j14A6^* | □☒☒□ | 24160, 31092, 33703 | 111532, 111627 | 37715, 108122 |
| 111604 | *CG5446^KG06435^* | □☒☒□ | 7516, 40824 |  | 41797, 108207 |
| 111602 | *CG15141^KG06005^* | □☒☒□ | 7838 |  | 19307, 108728 |
| 111594 | *CG42327^KG05924^* | □☒☒□ | 7960, 33356, 52997 |  | 39841, 100914, 106630 |
| 111424 | *TkR99D^s2222^* | □☒☒□ | 7691, 27513, 55732 |  | 1372, 1374, 44369 |
| **Validation methods:** a, the P-insertion lines have been assessed previously as mutants (information from FlyBase); b, complementation tests using other lethal alleles of the P-insertion genes or a deficiency covering the locus of the P-insertion genes; c, RNAi-based knockdown of P-insertion genes; d, MARCM clones generated for secondary lethal alleles. □ not examined; ■ examined, positive result; ☒ examined, negative result. * *UAS-RNAi* lines for P-insertion genes crossed to *ppk-GAL4 UAS-mCD8-GFP* flies exhibiting dendritic defects of c4da neurons. | | | | | |

**Table S3. List of P-insertion strains showing normal dendritic morphologies in our screen**

|  | **DGRC #** | **Gene** |  | **DGRC #** | **Gene** |  | **DGRC #** | **Gene** |  | **DGRC #** | **Gene** |  |
| --- | --- | --- | --- | --- | --- | --- | --- | --- | --- | --- | --- | --- |
| 1 | 111003 | *Neos* | 58 | 111183 | *jeb* | 115 | 111407 | *neur* | 172 | 111580 | *Rat1* |  |
| 2 | 111008 | Unknown | 59 | 111195 | *CG13438* | 116 | 111408 | *Pp1-87B* | 173 | 111587 | *PIG-U* |  |
| 3 | 111009 | *nmo* | 60 | 111197 | *CG11030* | 117 | 111410 | Unknown | 174 | 111589 | *vih* |  |
| 4 | 111014 | *l(3)L0539* | 61 | 111198 | *Cdk4* | 118 | 111415 | *eff* | 175 | 111590 | *IP3K1* |  |
| 5 | 111016 | Unknown | 62 | 111208 | *hoip* | 119 | 111418 | *vib* | 176 | 111595 | *γCOP* |  |
| 6 | 111019 | Unknown | 63 | 111210 | *Sema2b* | 120 | 111427 | *nero* | 177 | 111598 | *ND-51* |  |
| 7 | 111022 | *l(3)76BDm* | 64 | 111216 | *AsnRS-m* | 121 | 111428 | *awd* | 178 | 111607 | *Nf-YA* |  |
| 8 | 111024 | Unknown | 65 | 111224 | *wcd* | 122 | 111429 | *dbe* | 179 | 111608 | *mRpL17* |  |
| 9 | 111025 | *mRpS28* | 66 | 111226 | *Psc* | 123 | 111431 | *mol* | 180 | 111610 | *chinmo* |  |
| 10 | 111026 | *kra* | 67 | 111237 | *dom* | 124 | 111435 | *mRpL4* | 181 | 111611 | *CG8108* |  |
| 11 | 111028 | Unknown | 68 | 111238 | *AGO1* | 125 | 111436 | Unknown | 182 | 111615 | *aop* |  |
| 12 | 111032 | Unknown | 69 | 111247 | *THG* | 126 | 111438 | *Opa1* | 183 | 111616 | *CtBP* |  |
| 13 | 111035 | *Csk* | 70 | 111248 | *prod* | 127 | 111441 | Unknown | 184 | 111618 | *pbl* |  |
| 14 | 111036 | Unknown | 71 | 111251 | *l(2)k01209* | 128 | 111443 | *eIF6* | 185 | 111622 | *crp* |  |
| 15 | 111038 | *B52* | 72 | 111255 | *FKBP59* | 129 | 111448 | *Nop60B* | 186 | 111624 | *CG3347* |  |
| 16 | 111040 | *Hsc70-4* | 73 | 111256 | Unknown | 130 | 111452 | *Nurf-38* | 187 | 111626 | *aph-1* |  |
| 17 | 111045 | *RpL30* | 74 | 111258 | Unknown | 131 | 111456 | Unknown | 188 | 111627 | *trx* |  |
| 18 | 111048 | *mod(mdg4)* | 75 | 111263 | *Rs1* | 132 | 111457 | *Idh* | 189 | 111628 | *G6P* |  |
| 19 | 111049 | *Dph5* | 76 | 111271 | *RpL30* | 133 | 111462 | Unknown | 190 | 111633 | *ttk* |  |
| 20 | 111050 | *Gclm* | 77 | 111275 | *Kebab* | 134 | 111463 | Unknown | 191 | 111636 | *CG4382* |  |
| 21 | 111065 | *PCNA* | 78 | 111276 | *Gmd* | 135 | 111464 | Unknown | 192 | 111641 | *CG18477* |  |
| 22 | 111069 | *Mam* | 79 | 111278 | *ND-PDSW* | 136 | 111465 | Unknown | 193 | 111644 | Unknown |  |
| 23 | 111070 | *Drk* | 80 | 111283 | *mir-14* | 137 | 111467 | Unknown | 194 | 111655 | Unknown |  |
| 24 | 111072 | *Hrb27C* | 81 | 111292 | *Coprox* | 138 | 111473 | Unknown | 195 | 111656 | *kis* |  |
| 25 | 111074 | *Shg* | 82 | 111293 | *bic* | 139 | 111477 | Unknown | 196 | 111662 | *Rack1* |  |
| 26 | 111079 | *Crol* | 83 | 111302 | Unknown | 140 | 111488 | *Hipk* | 197 | 111663 | *ppk5* |  |
| 27 | 111081 | Unknown | 84 | 111310 | *Hel25E* | 141 | 111492 | *mnd* | 198 | 111669 | *Fgop2* |  |
| 28 | 111083 | *Neb* | 85 | 111312 | *ttv* | 142 | 111498 | *ND-MWFE* | 199 | 111672 | *osp* |  |
| 29 | 111087 | *Tfb1* | 86 | 111317 | *Dad1* | 143 | 111501 | *mam* | 200 | 111675 | *Pgk* |  |
| 30 | 111091 | *S* | 87 | 111319 | *Lis-1* | 144 | 111506 | *Prp8* | 201 | 111685 | *CG4896* |  |
| 31 | 111095 | *Acsl* | 88 | 111323 | *CG8026* | 145 | 111512 | *CG3793* | 202 | 111686 | *CG13928* |  |
| 32 | 111096 | Unknown | 89 | 111334 | *Cyt-c-p* | 146 | 111513 | *CycE* | 203 | 111689 | *RpL10Ab* |  |
| 33 | 111097 | *Nmd* | 90 | 111335 | *eIF3j* | 147 | 111516 | *Kr-h1* | 204 | 111692 | *Blimp-1* |  |
| 34 | 111099 | *Sema2a* | 91 | 111336 | *barr* | 148 | 111517 | *CG7728* | 205 | 111694 | *CG10336* |  |
| 35 | 111106 | *Tutl* | 92 | 111339 | *Nrk* | 149 | 111522 | *Mys45A* | 206 | 111699 | *mib2* |  |
| 36 | 111108 | *Tai* | 93 | 111340 | *Pen* | 150 | 111525 | *VhaAC45* | 207 | 111701 | Unknown |  |
| 37 | 111109 | *TER94* | 94 | 111342 | *Egm* | 151 | 111527 | *Pabp2* | 208 | 111705 | *muc* |  |
| 38 | 111111 | *RpS21* | 95 | 111351 | *cos* | 152 | 111528 | *Pbp49* | 209 | 111706 | *nesd* |  |
| 39 | 111112 | *Grh* | 96 | 111356 | *ebi* | 153 | 111530 | *AspRS* ^a^ | 210 | 111707 | *VhaSFD* |  |
| 40 | 111113 | *Vha68-2* | 97 | 111358 | *cnk* | 154 | 111532 | *trx* | 211 | 111709 | *CG13694* |  |
| 41 | 111118 | *Btk29A* | 98 | 111361 | *emb* | 155 | 111533 | *cg* | 212 | 111711 | *bun* |  |
| 42 | 111119 | *AGO1* | 99 | 111363 | *Cpsf160* | 156 | 111534 | *Vha44* | 213 | 111712 | *ND-B14.5B* |  |
| 43 | 111121 | *nop5* | 100 | 111366 | *Reph* | 157 | 111536 | *nuf* | 214 | 111716 | Unknown |  |
| 44 | 111122 | *vkg* | 101 | 111369 | *Tor* | 158 | 111538 | *Cul4* | 215 | 111719 | *pim* |  |
| 45 | 111130 | *Stip1* | 102 | 111370 | *Tapδ* | 159 | 111542 | *sls* | 216 | 111723 | *CG6700* |  |
| 46 | 111134 | *ed* | 103 | 111371 | *fdl* | 160 | 111545 | *Tsf2* | 217 | 111725 | Unknown |  |
| 47 | 111141 | *tkv* | 104 | 111372 | Unknown | 161 | 111548 | *sti* | 218 | 111726 | Unknown |  |
| 48 | 111143 | *eIF4A* | 105 | 111375 | *PCNA* | 162 | 111552 | *pnt* | 219 | 111729 | Unknown |  |
| 49 | 111144 | *Rpp30* | 106 | 111377 | *HnRNP-K* | 163 | 111556 | *PGRP-LF* | 220 | 111731 | *Cse1* |  |
| 50 | 111147 | Unknown | 107 | 111381 | Unknown | 164 | 111557 | *SmE* | 221 | 111733 | *tara* |  |
| 51 | 111149 | *ptc* | 108 | 111382 | *smid* | 165 | 111558 | *Dlg5* | 222 | 111735 | *Sec24CD* |  |
| 52 | 111150 | *lolal* | 109 | 111384 | Unknown | 166 | 111560 | *l(2)35Cc* | 223 | 111736 | Unknown |  |
| 53 | 111160 | *dup* | 110 | 111386 | Unknown | 167 | 111564 | *Lar* | 224 | 111737 | Unknown |  |
| 54 | 111161 | Unknown | 111 | 111389 | *vers* | 168 | 111566 | *Baldspot* | 225 | 114341 | *snRNA:U6atac* |  |
| 55 | 111163 | *Mtor* | 112 | 111391 | *btl* | 169 | 111572 | *Cbl* | 226 | 114349 | *Ehbp1* |  |
| 56 | 111164 | Trs23 | 113 | 111395 | *RhoGAP71E* | 170 | 111577 | *CG31694* | 227 | 114488 | *sds22* |  |
| 57 | 111176 | *spt4* | 114 | 111398 | *l(3)72Dn* | 171 | 111578 | *Arpc2* | 228 | 114534 | *Smu1* |  |
| ^a^ *AspRS^KG03912^* (DGRC #111530) failed to complement with another lethal allele *AspRS^1^*; *UAS-AspRS RNAi* lines (VDRC # 7750 and BDSC# 42606) were crossed to *ppk-GAL4 UAS-mCD8-GFP* flies, and their dendritic morphologies were indistinguishable from the control. | | | | | | | | | | | | |
